# Origin and Post-Glacial Dispersal of Mitochondrial DNA Haplogroups C and D in Northern Asia

**DOI:** 10.1371/journal.pone.0015214

**Published:** 2010-12-21

**Authors:** Miroslava Derenko, Boris Malyarchuk, Tomasz Grzybowski, Galina Denisova, Urszula Rogalla, Maria Perkova, Irina Dambueva, Ilia Zakharov

**Affiliations:** 1 Institute of Biological Problems of the North, Russian Academy of Sciences, Magadan, Russia; 2 The Nicolaus Copernicus University, Ludwik Rydygier Collegium Medicum, Institute of Forensic Medicine, Department of Molecular and Forensic Genetics, Bydgoszcz, Poland; 3 Institute of General and Experimental Biology, Russian Academy of Sciences, Ulan-Ude, Russia; 4 Vavilov Institute of General Genetics, Russian Academy of Sciences, Moscow, Russia; Natural History Museum of Denmark, Denmark

## Abstract

More than a half of the northern Asian pool of human mitochondrial DNA (mtDNA) is fragmented into a number of subclades of haplogroups C and D, two of the most frequent haplogroups throughout northern, eastern, central Asia and America. While there has been considerable recent progress in studying mitochondrial variation in eastern Asia and America at the complete genome resolution, little comparable data is available for regions such as southern Siberia – the area where most of northern Asian haplogroups, including C and D, likely diversified. This gap in our knowledge causes a serious barrier for progress in understanding the demographic pre-history of northern Eurasia in general. Here we describe the phylogeography of haplogroups C and D in the populations of northern and eastern Asia. We have analyzed 770 samples from haplogroups C and D (174 and 596, respectively) at high resolution, including 182 novel complete mtDNA sequences representing haplogroups C and D (83 and 99, respectively). The present-day variation of haplogroups C and D suggests that these mtDNA clades expanded before the Last Glacial Maximum (LGM), with their oldest lineages being present in the eastern Asia. Unlike in eastern Asia, most of the northern Asian variants of haplogroups C and D began the expansion after the LGM, thus pointing to post-glacial re-colonization of northern Asia. Our results show that both haplogroups were involved in migrations, from eastern Asia and southern Siberia to eastern and northeastern Europe, likely during the middle Holocene.

## Introduction

The territory of northern Asia is of crucial importance for the study of early human dispersal and the peopling of the Americas. Recent findings about the peopling of northern Asia reconstructed by archaeologists suggest that modern humans colonized the southern part of Siberia around 40 thousand years ago (kya) and the far northern parts of Siberia and ancient Beringia, a prerequisite for colonization of the Americas, by approximately 30 kya [1], [2]. Current molecular genetic evidence suggest that the initial founders of the Americas emerged from an ancestral population of less than 5,000 individuals that evolved in isolation, likely in Beringia, from where they dispersed south after approximately 17 kya [3]–[6]. The genetic data have not revealed multiple late-Pleistocene migrations, but do distinguish a Holocene dispersal of Eskimo-Aleuts from northeastern Asia as well as detect two distinct almost concomitant paths for the Paleo-Indian dispersal from Beringia approximately 15–17 kya [7]. Additionally, the first successful genome sequencing of a 4,000-year-old Greenland individual belonging to the Saqqaq Culture has provided an unambiguously evidence for a close relationship between Saqqaq and Siberian Arctic populations and for migration from Siberia into the New World some 5.5 kya, independent of that giving rise to the modern Native Americans and Inuit [8].

Notably, most of genetic evidence concerning the peopling of Americas has been acquired from the analysis of the mtDNA haplogroups at the highest level of molecular resolution – that of complete mtDNA sequences [4]–[7], [9]–[12]. A comprehensive overview of all available complete mtDNA genomes has allowed reconstruction of the detailed phylogeny of the six Native American haplogroups (A2, B2, C1, D1, X2a, and D4h3), identification of their internal clades and candidate founder sequences, and estimation of their expansion times into the Americas [5], [7], [9]. Meanwhile, irrespective of their likely ancestral status relative to Native Americans, the northern Asian populations have been profoundly underrepresented in the published complete genome mtDNA data sets. To date, only few studies dealing with complete mtDNA variation in northern Asian populations have been published [10], [12], [13]. The study of Derenko et al. [10] focused mainly on southern Siberian mtDNA variation and provided evidence that the southern Siberian mtDNA pool harbors several lineages associated with the Late Upper Paleolithic and/or early Neolithic dispersals from both eastern Asia and western Eurasia. Additionally, it has been shown that southern Siberia is likely to be a geographical source for the last postglacial maximum spread of some haplogroups to northern Siberia. More recently, Volodko et al. [12] have considerably expanded their previous survey of mtDNA diversity in Commander Aleuts [13] via complete mtDNA sequencing of haplogroups A, C and D dominating in northeastern Eurasian populations (Nganasan, Yukaghir, Chuvantsi, Chukchi, Siberian Eskimos). Results of these studies are undoubtedly useful for northern Asian mtDNA phylogeny reconstruction, but uncovering all of the most basal variation in the northern Asian mtDNA haplogroups will require major sampling and sequencing efforts with focusing on as much as possible diverse set of Siberian aboriginal populations.

More than a half of the northern Asian pool of mtDNA is fragmented into a number of subclades of haplogroups C and D, two of the most frequent haplogroups throughout northern, eastern, central Asia and America. Previous studies have proposed that haplogroups C and D originated around 30–50 kya in eastern Asia, from where they subsequently expanded northwards to southern Siberia, and further deep into northern Asia and the Americas, and westwards along the Steppe Belt extending from Manchuria to Europe [14], [15]. It has been also shown that haplogroups C and D were strongly involved in the late-glacial expansions from southern China to northeastern India [16]. In addition, because of their high frequency and wide distribution, haplogroups C and D most likely participated in all subsequent episodes of putative gene flow in northern Eurasia. These include (i) the Paleolithic colonization of Siberia that is associated with the development of macroblade industries (40–30 kya), (ii) further recolonization and possible replacement of early Siberians by microblade-making human populations from the Lake Baikal, Yenisei River, and Lena River basin regions (20 kya), (iii) appearance of pottery-making Neolithic tradition in the forest-steppe belt of northern Eurasia starting at about 14.5 kya and its expanding into the East European Plane (7 kya), (iv) the Neolithic dispersal of agriculture in eastern Asia, (v) the expansion of the Afanasievo and Andronovo cultures (5–3 kya), and (vi) more recent events of gene flow to eastern and central Europe.

As a result, it is likely that the dissection of haplogroups C and D into subhaplogroups of younger age and more limited geographic and ethnic distributions might reveal previously unidentified spatial frequency patterns, which in turn could be correlated to prehistoric and historical migratory events. However, until now, haplogroups C and D have been resolved genealogically only partially allowing for the identification of seven principal subclades (C1, C4, C5, C7, D4, D5, D6) and some of their internal subclades, the phylogeography of which has been evaluated only in some instances [4], [5], [9], [10], [12], [13], [16]–[18].

To shed some light on the origin and dispersal of haplogroups C and D in Asia, we present here an analysis of the complete mtDNA genomes from populations distributed over the geographical range of these two haplogroups.

## Results and Discussion

### The spread of haplogroups C and D

Haplogroups C and D display an extremely wide geographic distribution and high frequencies over most of their range. Haplogroup C peaks over 50% among Yukaghirs of northeastern Asia, central Siberian Yakuts and Evenks as well as East-Sayan Tofalars. Its frequency is persistently above 20% in Altaian, West-Sayan and Baikal region populations and drops to 13% or less among Chukchis, Eskimos and Itelmens in the east, Altaian Kazakhs, Shors, and Oroks in the south, and Khants and Kets in the west. The diminishing line (frequencies under 5%) goes through the Turkic and Finno-Ugric populations of the Volga basin, further south through the populations of the Caucasus and western Asia. In the southern direction the decline of haplogroup C frequency is almost as sharp as in the west direction: it is very common in Mongolia (15%) and most of the populations of central Asia (7–18%), but occurs as rarely as 1–5% in Korea, China, Thailand, Japan, Island southeastern Asia and India. Haplogroup C is detected at a very low frequency in several populations of eastern and central Europe and virtually absent in western Europe and Africa (Table S1).

The second most common haplogroup in all northern Asian populations is haplogroup D, which is also very common in eastern, central Asia and America. Haplogroup D encompasses almost 20% of the total mtDNA variation in most of northern Asia and retains a very high overall frequency in all regional northern Asian groups (11–34%), central Asian (14–20%) and eastern Asian (10–43%) populations (Table S2). Its frequency declines towards the west and south, to 2% or less in India and western Asia, but in the Caucasus, Volga-Ural Region and southeastern Asia is still as high as 5–10%. Interestingly, haplogroup D is also found in some northeastern Europeans, like Karelians, Saami and Scandinavians, while haplogroup C is absent among them (Table S2).

### The phylogeny of haplogroup C

The phylogeny of the C sequences is illustrated in Figure S1. The average sequence divergence of the 174 C complete genome corresponds to a coalescence time estimate of 27.37 (19.55; 35.44) kya when using the sequence variation of the entire genome and 26.33±6.58 when only synonymous mutations are considered [19] (Table S3). The C tree shows an initial deep split into four sister subclades, C1, C4, C5 and C7, each containing several independent basal branches, one within C1, at least three within C4, four within C5, and three within C7 (Figure S1). The C1 branch is represented by C1a subclade which is a sister clade of the Native American subclades C1b, C1c, and C1d, which are dated to 18.6±2.3 kya [5], [9] and most likely arose early – either in Beringia or at a very initial stage of the Paleoindian southward migration [4]. The Asian C1a-branch derived likely from the same ancestral population as the three Native American subclades [4] shows a relatively lower coalescence time varying from 2 to 8.5 kya (1.97±1.97 kya for synonymous clock rate and 8.57 (2.6; 14.75) kya for complete mtDNA clock rate), implying that its expansion from Beringia occurred long after the end of the LGM.

The C4 branch shows a coalescence time of 20–22 kya, implying that it began to expand before the LGM. Inside haplogroup C4 a new subclade, C4e, specific for Altai region populations has been revealed (Figure S1). It is defined by transitions at nps 151, 152, 7307, 15479 and, together with Russian individual (Rus_184), characterized by lack of adenine insertion at np 2232, which is thought to be diagnostic for a whole subclade C4a'b'c [20]. This subclade represents a major fraction of C4 mtDNAs and can be further subdivided into C4a, C4b and Native American-specific branch C4c identified so far only in two Ijka-speakers from Colombia [4] and one Shuswap individual from British Columbia [21]. Cluster C4a dates to 19–25 kya, demonstrating the pre-LGM time of divergence, in contrast to C4b, which is characterized by younger coalescence time estimated as 6–7 kya.

The other major branch of the tree, C5 has a coalescence time of 14–17 kya, depending on the mutation rate used. The phylogeny of haplogroup C5 reveals at least four subhaplogroups (C5a-C5d) with the similar coalescence time estimates varying from 9 to 14 kya (Table S3). The C7 branch is the most ancient, with an estimated coalescence time of 26–28 kya, but in contrast to C4 and C5, which encompass the entire geographical range of C, C7 is present mainly in eastern Asian and northeastern Indian populations (Figure S1).

**Figure 1:**
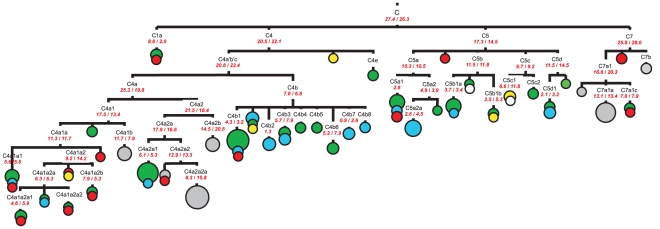
Complete mtDNA phylogenetic tree of haplogroup C. This schematic tree is based on phylogenetic tree presented in Figure S1. Time estimates (in kya) shown for mtDNA subclusters are based on the complete mtDNA genome clock (the first value) and the synonymous clock (the second value) [19]. The size of each circle is proportional to the number of individuals sharing the corresponding haplotype, with the smallest size corresponding to one individual. Geographic origin is indicated by different colors: northeastern Asian – in blue, central and southern Siberian – in green, eastern Asian – in red, Indian – in grey, European – in yellow, and others (*i.e.* of unknown population origin) – in white.

Based on complete mtDNA genome sequence information, we have identified several new subclusters within the C4 (C4a1a1, C4a1a2, C4a1a2b, C4b4, C4b5, C4b6, C4b8) and C5 (C5a2a1, C5b, C5b1a, C5c1, C5c2) subclades, as well as redefined some previously described clusters. Complete mtDNA sequence based phylogeographic analysis has shown a remarkable geographic distribution for some of haplogroup C subclusters (Figure 1). Thus, certain subclades of C4 and C5 were more prevalent in the southern Siberian populations being found mainly in Altai-Sayan and Baikal region populations (C4a1a1, C4a1a2b, C4b4, C4b5, C4b6), whereas others (C4b2, C4b7, C4b8, C5a2a) were found only in Arctic populations of Chukchi, Koryaks, Nganasans, and Yukaghirs. Interestingly, subclusters C4a1b, C4a2a2a, C4a2b, C4a2a2, and C7a1a encompass predominantly Indian mtDNA genomes, and show evolutionary ages within time frames of 8–20.5 kya. It is worth emphasizing that the ages of northern Asian clusters fall into the ranges of 3–14.5 kya, whereas the coalescence time estimates for Arctic region-specific lineages are not exceed 4.5 kya.

**Figure 2:**
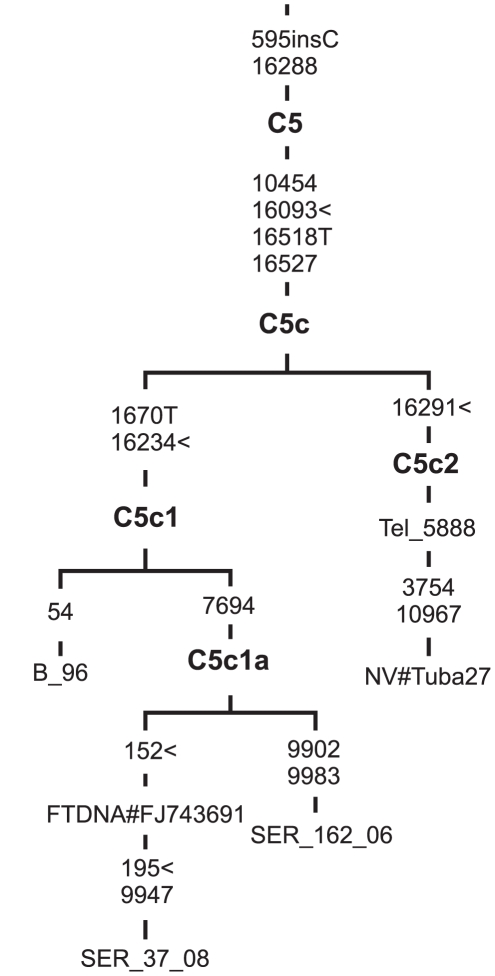
Complete mtDNA phylogenetic tree of subhaplogroup C5c. Numbers along links refer to substitutions scored relative to rCRS [51]. Transversions are further specified; ins denotes insertion of nucleotide; symbol < denotes parallel mutation. Subject origins are: Poles (B_96; Ser_37_08; Ser_162_06), Teleut (Tel), Tubalar (NV#Tuba from Volodko et al. [12]), and FamilyTreeDNA Project individual of unknown population origin (FTDNA).

Four of the new and two previously published sequences (one Teleut and one Tubalar from the Altai region of southern Siberia, three Poles from northern Poland, and one FamilyTreeDNA project individual of unknown ancestry) clustered into uncommon branch, named C5c, harboring the diagnostic motif 10454-16093-16518T-16527. Several mtDNAs with the same control-region motif were detected earlier at a low frequency in some European, Asian and southern Siberian populations – in Poles (0.4%), Belorussians (0.3%), Romanians (0.6%), Persians (0.2%), Kirghiz (1.1%), Altaians (0.9%), Teleuts (7.5%), Khakassians (0.9%) and Shors (4%) [4], [10], [22]–[28]. With the exception of mtDNAs from southern Siberia, which harbored additional control region transition at np 16291, all other C5c mtDNAs were characterized by another control region mutation at np 16234. The complete mtDNA genome phylogeny confirms that the C5c branch shows an initial split into two sister subclades, one encompassing mtDNAs from Europe (C5c1) and the other consisting of only two sequences from the Altai region of southern Siberia (C5c2) (Figure 2). It appears that European branch C5c1 is more differentiated, as far as two of three sequenced Polish mtDNAs formed a separate branch (C5c1a), defined by a coding region mutation at np 7694. The relatively large amount of internal variation accumulated in the Polish branch of C5c would mean that C5c1 arose *in situ* in Europe after the arrival of a C5c1 founder mtDNA from southern Siberia, and that C5c1 affiliation is a marker of maternal Siberian ancestry. The phylogeny depicted in Figure S1 provides additional information concerning the entry time of the founder mtDNA – the age of C5c node is estimated as 9.7 (3.17; 16.49) kya when using the sequence variation of the entire genome, and 9.2±4.74 when only synonymous mutations are considered (Table S3). The early presence of mtDNA lineages of eastern Asian ancestry in Europe is further confirmed by the discovery of a N9a haplotype in a Neolithic skeleton from the Szarvas site, located in southeastern Hungary that belonged to the Körös Culture, which appeared in eastern Hungary in the early 8th millennium B.P. [29].

### The phylogeny of haplogroup D

**Figure 3:**
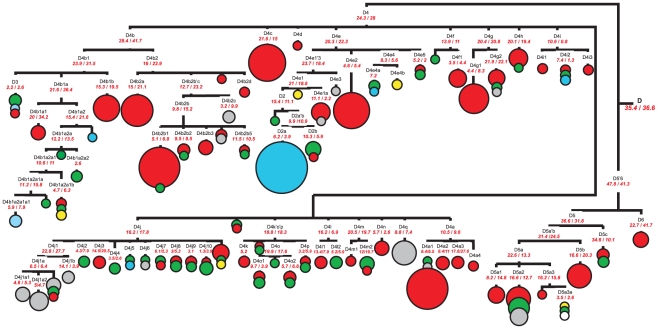
Complete mtDNA phylogenetic tree of haplogroup D. This schematic tree is based on phylogenetic tree presented in Figure S2. Time estimates (in kya) shown for mtDNA subclusters are based on the complete mtDNA genome clock (the first value) and the synonymous clock (the second value) [19]. The size of each circle is proportional to the number of individuals sharing the corresponding haplotype, with the smallest size corresponding to one individual. Geographic origin is indicated by different colors: northeastern Asian – in blue, central and southern Siberian – in green, eastern Asian – in red, Indian – in grey, European – in yellow, and others (*i.e.* of unknown population origin) – in white.

Haplogroup D has a likely pre-LGM time depth characterized by an overall coalescence time estimate of 35–37 kya, depending on the mutation rate used. Two of its major subclades, D4 and D6, have a similar age of 24–28 kya and 23–42 kya, respectively, whereas subclade D5 has an older coalescence time estimated as 32–37 kya (Table S4). Haplogroup D4, the most represented of D clades, is further subdivided into fifteen principal subclades (D4a–D4j, D4k'o'p', D4l–D4n, D4q), which range from ∼6 to ∼28 kya when using the sequence variation of the entire genome and from ∼3 to ∼42 kya when only synonymous mutations are counted. Some of these subclades have a very distinctive geographic distribution, which is highly informative about the demographic history of the northern Asia. Whilst all subclades are found in eastern Asia, so that eastern Asian lineages occur throughout the tree, few of them are specific for northern Asian populations (Figure 3).

**Figure 4:**
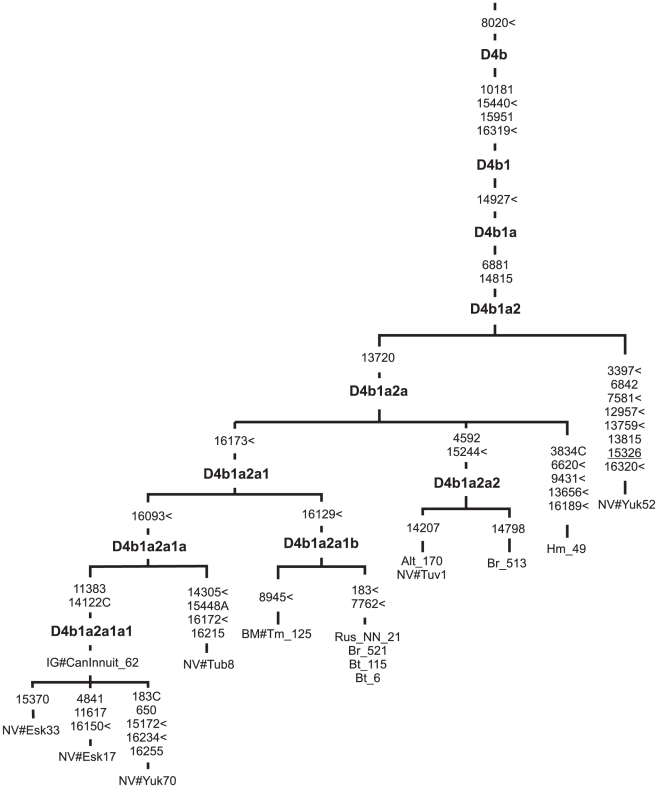
Complete mtDNA phylogenetic tree of subhaplogroup D4b1a2. Numbers along links refer to substitutions scored relative to rCRS [51]. Transversions are further specified; symbol < denotes parallel mutation, back mutation is underlined. Subject origins are: Russian (Rus), Buryat (Br), Barghut (Bt), Altaian (Alt), Khamnigan (Hm), Tuvinian (NV#Tuv from Volodko et al. [12], Yukaghir (NV#Yuk from Volodko et al. [12]), Tubalar (NV#Tub from Volodko et al. [12]), Eskimo (NV#Esk from Volodko et al. [12]), Canadian Inuit (IG#CanInnuit from Ingman, Gyllensten [32]), and Tatar (BM#Tm from Malyarchuk et al. [58]).

Of the subclades shared with eastern Asians, D4b1a, which is 21.62 (12.72; 30.88) or 36.42±10.85 ky old, falls into two branches, one of which, D4b1a2, is largely restricted to northern Asia (Figure 4). It should be noted that its major subclade, D4b1a2a, resulted from the earliest split from the Yukaghir mtDNA within D4b1a2, was described for the first time in Volodko et al. [12] and designated there as D3a2a. Based on the pattern of its geographic distribution and coalescence time estimated from six complete mtDNAs as 11.1±4.3 kya, the authors postulated a separate Upper Paleolithic migration initiated northward from the Altai-Sayan region of southern Siberia. Interestingly, the addition of a large set of completely sequenced mtDNAs from northern Asian populations has allowed us to refine the D4b1a2a phylogeny and subdivide it into at least two subclades - D4b1a2a1 and D4b1a2a2, and a rarer branch represented so far by a single mtDNA found in Khamnigans (Figure 4, Figure S2). The phylogeny based on the complete genome shows that D4b1a2a1 can be further subdivided into D4b1a2a1a, largely restricted to northeastern Asia and northern America, though extending occasionally to southern Siberia, and D4b1a2a1b found in eastern Asia (Barghuts), southern Siberia (Buryats) and eastern Europe (Russians, Tatars). To shed some light on the origin of D4b1a2a1, we surveyed almost 30,000 subjects from 116 population samples for the presence of the HVS1 motifs diagnostic for D4b1a2a1a (16093-16173-16223-16319-16362) and D4b1a2a1b (16129-16173-16223-16319-16362). The results of this survey are reported in Table S5. As can be seen, several HVS1 mtDNAs identical to those detected in Siberian Eskimos, Greenland and Canadian Inuit are distributed also among eastern European Kalmyks and Bashkirs as well as central Asian Karakalpaks. Moreover, similar HVS1 sequences occur both in northeastern Asia (Chukchi, Siberian Eskimo, Chuvantsi) and southern Siberia/central Asia (Altaians, Shors, Khakassians, Tubalars, Kirghiz, Uighurs) (Table S5). In contrast, D4b1a2a1b mtDNAs with different, but related, control region motifs were identified in southern Siberia/eastern Asia (Buryats, Barghuts, Khamnigans, Mongolians) and eastern Europe (Tatars, Maris, Bashkirs, Udmurts, Russians, Belorussians, Poles), but not in northeastern Asia and northern America. Thus, the phylogeography of its major subclusters implies that D4b1a2a1 arose in southern Siberia and dispersed fairly recently northward to northern Asia and America, and westward to eastern Europe. Furthermore, the second major subclade of D4b1a2a, D4b1a2a2 is only present in southern Siberia, also suggesting an origin in this region. It is striking that the sequence divergence of two major subclades of D4b1a2a was relatively small, corresponding to only 3–11 kya, thus implying a Holocene origin and expansion of these lineages in northern Eurasia. However, the age of D4b1a2 is estimated as 15 kya (using the complete mtDNA rate) and 21 kya (synonymous rate) pointing to a LGM/post LGM origin, and apparently before the Holocene origin of this subcluster (Table S4).

From the coalescence analysis it is evident that besides D4b1a2, only two other clusters bear the strongest signal for the post LGM expansion in northern Asia. Subclusters D4m2 and D2 demonstrate a coalescence age of 12–20 kya and 11–15 kya, respectively, which are comparable with the age of D4b1a2. It is also remarkable that within D4m2, an Altaian branch precedes subcluster D4m2a, which is characteristic for a broad range of Arctic, Subarctic and southern Siberian populations (Figure S2). Another D4 subcluster, D2, has its most likely homeland in the Baikal region of southern Siberia, from where it expanded in the Holocene northward to northeastern Asia and further to northern America. The remaining northern Asian-specific clusters of haplogroup D are significantly younger with the age estimates not exceeding 5–8 kya (Figure 3). Among these, subclusters D4e4a and D4l2 are characterized by prevalence in the Subarctic and Arctic regions, being found mostly in Evenks and Yukaghirs, whereas several newly described subclusters within haplogroup D4j (D4j4, D4j5, D4j7, D4j8, D4j9, D4j10) demonstrate more southern geographic distribution, being detected in a variety of southern Siberian populations (Figure S2).

It should be noted that the rare subcluster D4e4b has been detected in eastern Europe (in Tatars and Russians), thus pointing to a limited maternal gene flow between eastern Asia/southern Siberia and eastern Europe. One more mtDNA subcluster which may be indicative of eastern Asian influx into gene pool of eastern Europeans has been revealed in haplogroup D5a. It has been shown earlier that D5a mtDNAs, with the specific control region motif 16126-16136-16360, are present at a very low frequency in several populations of northeastern Europe (Saami, Karelians, Finns, Estonians, Komi, Russians of Arkhangelsk and Novgorod regions) as well as in central Asian Tajiks and Siberian Altaians and Mansi [10], [27], [30], [31]. Analysis of complete mtDNA phylogeny indicates that these mtDNAs belong to subhaplogroup named D5a3 defined by the only transition at np 16360 (Figure S2). It is obvious that mitochondrial genomes of Russian, Mansi and FamilyTreeDNA project individual belong to D5a3a branch harboring the entire HVS1 motif, whereas Korean mtDNA represents another D5a3 branch. In fact, this most ancestral sequence indicates that D5a3 lineages could have probably arise in eastern Asia about 16 kya, and that the other lineages, belonging to the D5a3a subgroup participated in a more recent European expansion around 2.6–3.5 kya (Figure 3). It should be noted that dispersal of Saami-specific Z1a mtDNAs shared a common ancestry with lineages from the Volga-Ural region as recently as ∼3 kya probably chronicles the same expansion [32].

### Conclusions

The peopling of northern Asia by anatomically modern humans probably began more than 40 kya, with the first evidence in the Altai region, suggesting the southern mountain belt of Siberia and Middle Siberian plateau as a likely route for this pioneer settlement of northern Asia [33]–[36]. The present-day variation of haplogroups C and D suggests that these mtDNA clades had already expanded before the LGM, with their oldest lineages being present in the eastern Asia. In particular, most of the eastern Asian subclades of haplogroup D show coalescence ages of between 15 and 42 kya, thus suggesting that some of them were already present here before the LGM. As for northern Asia, most of the present-day southern and northeastern Siberian variants of haplogroups C and D started to expand after the LGM. This can be partially ascribed, as in Europe [31], [37]–[39] and southeastern Asia [40], to the (re)colonization processes of areas which were unsuitable for human occupation during the LGM due to aridity and lower temperatures. The Late Glacial re-expansion of microblade-making populations from the small refugial areas in southern Yenisei and Transbaikal region of southern Siberia at the end of the Ice Age from ∼18 kya could be suggested as a major demographic process signaled in the mtDNA by the distribution of northern Asian-specific subclades of haplogroups C and D. The age of haplogroup C5, ∼14–17 kya, supports this postulated arrival after the LGM, as does the age of the D2 and D4b1a2, which date to ∼11–21 kya. However, all northeastern Asian-specific subclades present ages lower than 10 kya, so it is possible that their arrival into the Arctic region of northern Asia occurred later, in Holocene.

Importantly, we have not found in northern Asia any genetic signatures of sufficient antiquity to indicate traces of pre-LGM expansions, that originated from the Upper Paleolithic industries that were present both in the southern Siberia and Siberian Arctic, and that date back to ∼30 kya, well before the LGM [1], [34], [36]. Apparently, the Upper Paleolithic population of northern Asia did not leaving a genetic mark on the female lineages of modern Siberians. It is probable that the initial population expansion in the southern Siberia region involved maternal lineages other than haplogroups C and D. Nevertheless none of the remaining northern Asian haplogroups became as frequent in Siberia as haplogroups C and D.

## Materials and Methods

### Ethics Statement

The study was approved by Bioethics Committee of the Nicolaus Copernicus University in Torun, The Ludwik Rydygier Collegium in Bydoszcz, Poland (statements no. KB/32/2002 and KB/414/2008 from 28 January, 2002 and 17 September, 2008, respectively). All subjects provided written informed consent for the collection of samples and subsequent analysis.

### Mitochondrial genome sequencing

Out of about 4500 samples that had been screened previously for haplogroup-diagnostic RFLP markers and subjected to control region sequencing [10], [27], [41]–[49], a total of 182 samples representing haplogroups C (83 samples) and D (99 samples) were selected for complete mtDNA sequencing (Table S6). Samples were selected to include the widest possible range of haplogroups C and D internal variation based on mtDNA control region variability data. Complete mtDNA sequencing was performed using the methodology described in detail by Torroni et al. [50]. DNA sequence data were analyzed using SeqScape v. 2.5 software (Applied Biosystems) and compared with the revised Cambridge reference sequence (rCRS) [51].

### Phylogenetic analysis

For reconstruction of the phylogenies of haplogroups C and D, the data obtained in this study and those published previously [4], [10], [11]–[14], [16]–[18], [32], [52]–[61], as well as FamilyTreeDNA project data available at PhyloTree [62], were taken into account. A nomenclature, which we hereby update, follows Kong et al. [18] and van Oven and Kayser [62], with several new modifications.

The most-parsimonious trees of the complete mtDNA sequences were reconstructed manually, and verified by means of the Network 4.5.1.0 software [63], and using mtPhyl software (http://eltsov.org), which is designed to reconstruct maximum parsimony phylogenetic trees. Both applications calculate haplogroup divergence estimates (ρ) and their error ranges, as average number of substitutions in mtDNA clusters (haplogroups) from the ancestral sequence type [64]. Values of mutation rates based on mtDNA complete genome variability data (one mutation every 3624 years [19]) and synonymous substitutions (one mutation every 7884 years [19]) were used.

Overall, 770 mitochondrial genomes – 174 C and 596 D – were analyzed. Nucleotide positions (nps) showing point indels and transversions located between nps 16180-16193 and 303-315 were excluded from the phylogenetic analysis. The GenBank accession numbers for the complete mitochondrial genomes reported in this paper are FJ951438-FJ951618.

## Supporting Information

Figure S1 Phylogenetic tree of haplogroup C, constructed using the program mtPhyl. Numbers along links refer to substitutions scored relative to rCRS [51]. Transversions are further specified; ins and del denote insertions and deletions of nucleotides, respectively; back mutations are underlined; symbol < denotes parallel mutation. Sequences indicated in red print are new (Table S6) while the others have been taken from Ingman et al. [52]; Kong et al. [14]; Tanaka et al. [17]; Starikovskaya et al. [55]; Kong et al. [18]; Derenko et al. [10]; Ingman and Gyllensten [32]; Volodko et al. [12]; Chandrasekar et al. [16]; Malyarchuk et al. [58]. The particular sequences from these sources are referred to as MI, QK, MT, ES, QP, MD, IG, NV, AC, and BM respectively, followed by number sign (#) and the original sample code. Established haplogroup labels are shown in black; blue are redefined and red are newly identified haplogroups in the present study.
(XLS)

Figure S2 Phylogenetic tree of haplogroup D, constructed using the program mtPhyl. Numbers along links refer to substitutions scored relative to rCRS [51]. Transversions are further specified; ins and del denote insertions and deletions of nucleotides, respectively; back mutations are underlined; symbol < denotes parallel mutation. Sequences indicated in red print are new (Table S6) while the others have been taken from the literature (Ingman et al. [52]; Derbeneva et al. [13]; Kong et al. [14]; Mishmar et al. [61]; Tanaka et al. [17]; Macaulay et al. [54]; Starikovskaya et al. [55]; Kong et al. [18]; Derenko et al. [10]; Ingman and Gyllensten [32]; Tamm et al. [4]; Gilbert et al. [11]; Volodko et al. [12]; Chandrasekar et al. [16]; Hartmann et al. [56]; Malyarchuk et al. [58]; Nohira et al. [59]; Tabbada et al. [60]; Ueno et al. [57]) and FamilyTreeDNA project data available at PhyloTree.org [62]. The particular sequences from these sources are referred to as MI, OD, QK, DM, MT, VM, ES, QP, MD, IG, ET, MG, NV, AC, AH, BM, CN, KT, HU, and FTDNA respectively, followed by number sign (#) and the original sample code. Established haplogroup labels are shown in black; blue are redefined and red are newly identified haplogroups in the present study.
(XLSX)

Table S1 Population distribution and frequencies of haplogroup C and its subhaplogroups C1, C5 and C*.
(DOC)

Table S2 Population distribution and frequencies of haplogroup D and its subhaplogroups D2, D4 and D5.
(DOC)

Table S3 Age estimates of haplogroup C subclusters calculated using different mutation rates.
(DOC)

Table S4 Age estimates of haplogroup D subclusters calculated using different mutation rates.
(DOC)

Table S5 Distribution of D4b1a2a1 HVS1 mtDNA sequences in populations of northern Asia and America.
(DOC)

Table S6 Control-region variation of the completely sequenced mtDNAs belonging to haplogroups C and D.
(DOC)
