# Supplementary material for: Origin and Post-Glacial Dispersal of Mitochondrial DNA Haplogroups C and D in Northern Asia
Source: PLoS One. 2010 Dec 21;5(12):e15214. doi: 10.1371/journal.pone.0015214 (PMC3006427; doi:10.1371/journal.pone.0015214)
Supplement: Table S1 — Population distribution and frequencies of haplogroup C and its subhaplogroups C1, C5 and C*. (DOC) [file pone.0015214.s003.doc]

Table S1. Population distribution and frequencies of haplogroup C and its subhaplogroups C1, C5 and C*

| Region/Population | No. of subjects | Haplogroup frequency (%) | | | | References |
| --- | --- | --- | --- | --- | --- | --- |
|  |  | C | C1 | C* | C5 |  |
| **America:** | 601 | 25.3 | 25.0 | 0.3 | 0.0 | Tamm et al. 2007 |
| **Northern Asia:** | 4719 | 26.1 | 0.3 | 19.8 | 6.0 |  |
| **Northeastern Asia:** | 1222 | 22.8 | 0.0 | 14.9 | 7.9 |  |
| Itelmens | 46 | 13.0 | 0.0 | 0.0 | 13.0 | Schurr et al. 1999 |
| Koryaks | 182 | 36.8 | 0.0 | 21.4 | 15.4 | Derenko and Shields 1997; Schurr et al. 1999 |
| Chukchi | 417 | 13.2 | 0.0 | 6.5 | 6.7 | Starikovskaya et al. 1998; Derenko et al. 2007; Tamm et al. 2007; Volodko et al. 2008 |
| Evens | 191 | 37.7 | 0.0 | 26.2 | 11.5 | Derenko and Shields 1997; Tamm et al. 2007 |
| Eskimos | 254 | 0.8 | 0.0 | 0.8 | 0.0 | Starikovskaya et al. 1998; Tamm et al. 2007; Volodko et al. 2008 |
| Yukaghirs | 100 | 67.0 | 0.0 | 54.0 | 13.0 | Volodko et al. 2008 |
| Chuvantsi | 32 | 31.3 | 0.0 | 31.3 | 0 | Volodko et al. 2008 |
| **Central Siberia:** | 445 | 52.6 | 0.0 | 41.1 | 11.5 |  |
| Evenks | 71 | 71.8 | 0.0 | 57.7 | 14.1 | Starikovskaya et al. 2005 |
| East Evenks | 45 | 64.4 | 0.0 | 37.8 | 26.7 | Derenko et al. 2007 |
| West Evenks | 73 | 46.6 | 0.0 | 39.7 | 6.9 | Derenko et al. 2007 |
| Yakuts | 254 | 46.9 | 0.0 | 37.5 | 9.4 | Derenko and Shields 1997; Fedorova et al. 2003; Derenko et al. 2007 |
| **Far East:** | 440 | 12.3 | 2.0 | 7.7 | 2.6 |  |
| Nanais | 85 | 9.4 | 1.2 | 5.9 | 2.3 | Tamm et al. 2007 |
| Negidals | 33 | 15.2 | 0.0 | 9.1 | 6.1 | Starikovskaya et al. 2005 |
| Nivkhs | 56 | 0.0 | 0.0 | 0.0 | 0.0 | Starikovskaya et al. 2005 |
| Oroks | 61 | 11.5 | 11.5 | 0.0 | 0.0 | Bermisheva et al. 2005 |
| Tubalars | 72 | 19.4 | 0.0 | 16.7 | 2.7 | Starikovskaya et al. 2005 |
| Udegeys | 46 | 17.4 | 0.0 | 17.4 | 0 | Starikovskaya et al. 2005 |
| Ulchi | 87 | 13.8 | 1.1 | 6.9 | 5.8 | Starikovskaya et al. 2005 |
| **Altai Region:** | 422 | 20.1 | 0.0 | 16.8 | 3.3 |  |
| Altai-Kizhi | 90 | 32.2 | 0.0 | 23.3 | 8.9 | Derenko et al. 2007 |
| Altaian Kazakhs | 98 | 8.2 | 0.0 | 8.2 | 0.0 | Derenko 2009 |
| Altaians | 110 | 19.1 | 0.0 | 19.1 | 0.0 | Derenko et al. 2003 |
| Telenghits | 71 | 16.9 | 0.0 | 14.1 | 2.8 | Derenko et al. 2007 |
| Teleuts | 53 | 28.3 | 0.0 | 20.7 | 7.6 | Derenko et al. 2007 |
| **East-Sayan Region:** | 337 | 49.9 | 0.0 | 40.1 | 9.8 |  |
| Todjins | 48 | 47.9 | 0.0 | 33.3 | 14.6 | Derenko et al. 2003 |
| Tofalars | 58 | 62.1 | 0.0 | 53.5 | 8.6 | Derenko et al. 2003 |
| Tuvinians | 231 | 47.2 | 0.0 | 38.1 | 9.1 | Derenko et al. 2000; 2003; 2007 |
| **West-Sayan Region:** | 192 | 20.8 | 0.0 | 19.3 | 1.5 |  |
| Khakassians | 110 | 27.3 | 0.0 | 25.5 | 1.8 | Derenko et al. 2003; 2007 |
| Shors | 82 | 12.2 | 0.0 | 11.0 | 1.2 | Derenko et al. 2007 |
| **Baikal Region:** | 807 | 19.0 | 0.5 | 14.3 | 4.2 |  |
| Barghuts | 149 | 20.1 | 0.0 | 16.7 | 3.4 | Derenko 2009 |
| Buryats | 419 | 21.2 | 0.7 | 15.7 | 4.8 | Derenko et al. 2000; 2003; 2007 |
| Kalmyks | 110 | 10.9 | 0.9 | 7.3 | 2.7 | Derenko et al. 2007 |
| Sojots | 30 | 20.0 | 0.0 | 16.7 | 3.3 | Derenko et al. 2003 |
| Khamnigans | 99 | 16.2 | 0.0 | 11.1 | 5.1 | Derenko et al. 2007 |
| **Western Siberia:** | 854 | 25.6 | 0.0 | 20.8 | 4.8 |  |
| Dolgans | 157 | 38.9 | 0.0 | 34.4 | 4.5 | Tamm et al. 2007 |
| Kets | 104 | 12.5 | 0.0 | 12.5 | 0.0 | Derbeneva et al. 2002a; Tamm et al. 2007 |
| Mansi | 161 | 18.6 | 0.0 | 16.8 | 1.8 | Derbeneva et al. 2002b; Pimenoff et al. 2008 |
| Khants | 106 | 10.4 | 0.0 | 7.6 | 2.8 | Pimenoff et al. 2008 |
| Nganasans | 78 | 33.3 | 0.0 | 15.4 | 17.9 | Derbeneva et al. 2002a; Goltsova et al. 2005 |
| Nenets | 58 | 31.0 | 0.0 | 25.9 | 5.1 | Saillard et al. 2000a |
| Nenets Tundra | 70 | 48.6 | 0.0 | 34.3 | 14.3 | Tamm et al. 2007 |
| Selkups | 120 | 21.6 | 0.0 | 20.8 | 0.8 | Tamm et al. 2007 |
| **Eastern Asia:** | 5166 | 3.6 | 0.1 | 2.7 | 0.8 |  |
| Ainu | 51 | 0.0 | 0.0 | 0.0 | 0.0 | Horai et al. 1996 |
| Koreans | 1297 | 2.0 | 0.0 | 1.1 | 0.1 | Horai et al. 1996; Tanaka et al. 2004; Lee et al. 2006; Derenko et al. 2007 |
| Mongolians | 150 | 15.3 | 1.3 | 12.0 | 2.0 | Kolman et al. 1998; Derenko et al. 2007 |
| Japanese | 1312 | 0.5 | 0.3 | 0.1 | 0.1 | Tanaka et al. 2004 |
| Daurs, China | 45 | 6.6 | 2.2 | 4.4 | 0.0 | Kong et al. 2003 |
| Kazakhs, China | 53 | 13.2 | 0.0 | 9.4 | 3.8 | Yao et al. 2004 |
| Chinese | 1930 | 4.5 | 0.0 | 3.7 | 0.8 | Horai et al. 1996; Kivisild et al. 2002; Yao et al. 2002; 2003; Metspalu et al. 2004; Wen et al. 2004 |
| Koreans, China | 48 | 0.0 | 0.0 | 0.0 | 0.0 | Kong et al. 2003 |
| Mongolians, China | 97 | 8.3 | 0.0 | 8.3 | 0.0 | Kong et al. 2003; Yao et al. 2004 |
| Orochens, China | 44 | 29.5 | 0.0 | 20.4 | 9.1 | Kong et al. 2003 |
| Uighurs, China | 47 | 6.4 | 0.0 | 6.4 | 0.0 | Yao et al. 2004 |
| Hui, China | 45 | 2.2 | 0.0 | 2.2 | 0.0 | Yao et al. 2004 |
| Evenks, China | 47 | 19.1 | 0.0 | 14.9 | 4.2 | Kong et al. 2003 |
| **Southeastern Asia:** | 1481 | 1.8 | 0.0 | 1.8 | 0.0 |  |
| Thai | 552 | 3.4 | 0.0 | 3.4 | 0.0 | Matspalu et al. 2004 |
| Island Southeastern Asia | 929 | 0.5 | 0.0 | 0.5 | 0.0 | Hill et al. 2007 |
| **Central Asia:** | 1430 | 10.3 | 0.3 | 7.8 | 2.2 |  |
| Kazakhs | 511 | 8.6 | 0.8 | 6.3 | 1.5 | Comas et al. 1998; Chaix et al. 2007; Tamm et al. 2007 |
| Karakalpaks | 108 | 14.8 | 0.0 | 13.0 | 1.8 | Chaix et al. 2007 |
| Kirghiz | 200 | 14.0 | 0.5 | 9.0 | 4.5 | Comas et al. 1998; Tamm et al. 2007 |
| Shugnan, Tajikistan | 44 | 18.2 | 0.0 | 18.2 | 0.0 | Quintana-Murci et al. 2004 |
| Tajiks | 82 | 7.3 | 0.0 | 7.3 | 0.0 | Derenko et al. 2007; Tamm et al. 2007 |
| Turkmens | 178 | 13.5 | 0.0 | 7.9 | 5.6 | Malyarchuk 2002; Quintana-Murci et al. 2004; Chaix et al. 2007 |
| Uzbeks | 130 | 6.1 | 0.0 | 4.6 | 1.5 | Quintana-Murci et al. 2004; Chaix et al. 2007 |
| Uighurs | 177 | 7.3 | 0.0 | 7.3 | 0.0 | Comas et al. 1998; Tamm et al. 2007 |
| **Western Asia:** | 1060 | 1.2 | 0.0 | 0.9 | 0.3 |  |
| Turks | 218 | 1.8 | 0.0 | 1.3 | 0.5 | Richards et al. 2000 |
| Kurdish | 135 | 0.7 | 0.0 | 0.0 | 0.7 | Quintana-Murci et al. 2004; Nasidze et al. 2005; Derenko et al. 2007 |
| Pakistani | 189 | 2.1 | 0.0 | 2.1 | 0.0 | Metspalu et al. 2004; Quintana-Murci et al. 2004 |
| Persians | 518 | 0.8 | 0.0 | 0.6 | 0.2 | Metspalu et al. 2004; Derenko et al. 2007 |
| **Southern Asia:** |  |  |  |  |  |  |
| India | 2544 | 1.2 | 0.0 | 1.2 | 0.0 | Metspalu et al. 2004 |
| **Europe:** | 2783 | 0.8 | 0.0 | 0.4 | 0.4 |  |
| Hungarians | 344 | 0.6 | 0.0 | 0.6 | 0.0 | Lahermo et al. 2000; Egyed et al. 2007 |
| Poles | 947 | 0.5 | 0.0 | 0.0 | 0.5 | Richards et al. 2000; Malyarchuk et al. 2002; Grzybowski et al. 2007 |
| Romanians | 360 | 2.8 | 0.0 | 1.7 | 1.1 | Egyed et al. 2007 |
| Russians | 1132 | 0.5 | 0.0 | 0.3 | 0.2 | Malyarchuk 2002; Orekhov et al. 1999; Richards et al. 2000; Malyarchuk and Derenko 2001; Malyarchuk et al. 2002; 2004; Belyaeva et al. 2003; Grzybowski et al. 2007 |
| **Caucasus:** | 829 | 5.6 | 0.0 | 5.1 | 0.5 |  |
| Azerbaijanians | 89 | 3.4 | 0.0 | 3.4 | 0.0 | Richards et al. 2000; Nasidze and Stoneking 2001 |
| Georgians | 57 | 1.8 | 0.0 | 0.0 | 1.8 | Nasidze and Stoneking 2001 |
| Darginians | 37 | 2.7 | 0.0 | 2.7 | 0.0 | Nasidze and Stoneking 2001 |
| Kabardinians | 51 | 2.0 | 0.0 | 2.0 | 0.0 | Nasidze and Stoneking 2001 |
| Northern Caucasians | 199 | 4.0 | 0.0 | 4.0 | 0.0 | Richards et al. 2000 |
| Nogays | 206 | 12.1 | 0.0 | 10.7 | 1.4 | Bermisheva et al. 2005 |
| Osettins | 123 | 3.3 | 0.0 | 3.3 | 0.0 | Nasidze et al. 2004; 2005 |
| Chechens | 67 | 4.5 | 0.0 | 4.5 | 0.0 | Nasidze and Stoneking 2001 |
| **Volga- Ural Region:** | 1323 | 3.7 | 0.2 | 3.5 | 0.0 |  |
| Bashkirs | 207 | 12.1 | 1.5 | 10.6 | 0.0 | Bermisheva et al. 2002 |
| Komis | 121 | 5.0 | 0.0 | 5.0 | 0.0 | Bermisheva et al. 2002 |
| Maris | 234 | 0.9 | 0.0 | 0.9 | 0.0 | Bermisheva et al. 2002; Orekhov 2002 |
| Mordvins | 120 | 1.7 | 0.0 | 1.7 | 0.0 | Bermisheva et al. 2002 |
| Tatars | 310 | 2.3 | 0.0 | 2.3 | 0.0 | Bermisheva et al. 2002; Orekhov 2002 |
| Udmurts | 189 | 2.7 | 0.0 | 2.7 | 0.0 | Bermisheva et al. 2002 |
| Chuvashis | 142 | 1.4 | 0.0 | 1.4 | 0.0 | Bermisheva et al. 2002 |

Note. References for population data are as in the Table S5. The population subdivision of C sequences into subhaplogroups was performed based on specific HVS1/HVS2 motifs: 16325C-290delA-291delA for C1 and 16288C for C5. C* includes the remaining sequences with the exception of C1 and C5.
