# Supplementary material for: Origin and Post-Glacial Dispersal of Mitochondrial DNA Haplogroups C and D in Northern Asia
Source: PLoS One. 2010 Dec 21;5(12):e15214. doi: 10.1371/journal.pone.0015214 (PMC3006427; doi:10.1371/journal.pone.0015214)
Supplement: Table S2 — Population distribution and frequencies of haplogroup D and its subhaplogroups D2, D4 and D5. (DOC) [file pone.0015214.s004.doc]

Table S2. Population distribution and frequencies of haplogroup D and its subhaplogroups D2, D4 and D5

| Region/Population | No. of subjects | Haplogroup frequency (%) | | | | References |
| --- | --- | --- | --- | --- | --- | --- |
|  |  | D | D2 | D4 | D5 |  |
| **Northern Asia:** | 4719 | 19.6 | 1.7 | 16.7 | 1.2 |  |
| **Northeastern Asia:** | 1222 | 14.6 | 5.5 | 8.4 | 0.7 |  |
| Itelmens | 46 | 0.0 | 0.0 | 0.0 | 0.0 | Schurr et al. 1999 |
| Koryaks | 182 | 1.1 | 0.0 | 1.1 | 0.0 | Derenko and Shields 1997; Schurr et al. 1999 |
| Chukchi | 417 | 15.4 | 7.7 | 7.7 | 0.0 | Starikovskaya et al. 1998; Derenko et al. 2007; Tamm et al. 2007; Volodko et al. 2008 |
| Evens | 191 | 21.5 | 0.5 | 19.4 | 1.6 | Derenko and Shields 1997; Tamm et al. 2007 |
| Eskimos | 254 | 19.3 | 13.0 | 6.3 | 0.0 | Starikovskaya et al. 1998; Tamm et al. 2007; Volodko et al. 2008 |
| Yukaghirs | 100 | 19.0 | 0.0 | 14.0 | 5.0 | Volodko et al. 2008 |
| Chuvantsi | 32 | 9.4 | 3.1 | 6.3 | 0.0 | Volodko et al. 2008 |
| **Central Siberia:** | 445 | 14.2 | 0.5 | 11.9 | 1.8 |  |
| Evenks | 71 | 21.1 | 0.0 | 19.7 | 1.4 | Starikovskaya et al. 2005 |
| East Evenks | 45 | 24.4 | 2.2 | 22.2 | 0.0 | Derenko et al. 2007 |
| West Evenks | 73 | 30.1 | 0.0 | 23.3 | 6.8 | Derenko et al. 2007 |
| Yakuts | 256 | 5.9 | 0.4 | 4.7 | 0.8 | Derenko and Shields 1997; Fedorova et al. 2003; Derenko et al. 2007 |
| **Far East:** | 440 | 27.5 | 0.0 | 26.6 | 0.9 |  |
| Nanais | 85 | 25.9 | 0.0 | 25.9 | 0.0 | Tamm et al. 2007 |
| Negidals | 33 | 24.2 | 0.0 | 24.2 | 0.0 | Starikovskaya et al. 2005 |
| Nivkhs | 56 | 28.6 | 0.0 | 28.6 | 0.0 | Starikovskaya et al. 2005 |
| Oroks | 61 | 68.8 | 0.0 | 67.2 | 1.6 | Bermisheva et al. 2005 |
| Tubalars | 72 | 19.5 | 0.0 | 15.3 | 4.2 | Starikovskaya et al. 2005 |
| Udegeys | 46 | 0.0 | 0.0 | 0.0 | 0.0 | Starikovskaya et al. 2005 |
| Ulchi | 87 | 21.8 | 0.0 | 21.8 | 0.0 | Starikovskaya et al. 2005 |
| **Altai Region:** | 422 | 18.7 | 0.0 | 17.0 | 1.7 |  |
| Altai-Kizhi | 90 | 8.9 | 0.0 | 8.9 | 0.0 | Derenko et al. 2007 |
| Altaian Kazakhs | 98 | 26.5 | 0.0 | 22.5 | 4.0 | Derenko 2009 |
| Altaians | 110 | 15.4 | 0.0 | 13.6 | 1.8 | Derenko et al. 2003 |
| Telenghits | 71 | 21.1 | 0.0 | 21.1 | 0.0 | Derenko et al. 2007 |
| Teleuts | 53 | 24.5 | 0.0 | 22.6 | 1.9 | Derenko et al. 2007 |
| **East-Sayan Region:** | 337 | 11.3 | 0.9 | 8.6 | 1.8 |  |
| Todjins | 48 | 4.2 | 0.0 | 4.2 | 0.0 | Derenko et al. 2003 |
| Tofalars | 58 | 0.0 | 0.0 | 0.0 | 0.0 | Derenko et al. 2003 |
| Tuvinians | 231 | 15.6 | 1.3 | 11.7 | 2.6 | Derenko et al. 2000; 2003; 2007 |
| **West-Sayan Region:** | 192 | 13.5 | 0.0 | 12.5 | 1.0 |  |
| Khakassians | 110 | 14.5 | 0.0 | 13.6 | 0.9 | Derenko et al. 2003; 2007 |
| Shors | 82 | 12.2 | 0.0 | 11.0 | 1.2 | Derenko et al. 2007 |
| **Baikal Region:** | 807 | 34.2 | 1.1 | 30.9 | 2.2 |  |
| Barghuts | 149 | 35.6 | 2.0 | 32.9 | 0.7 | Derenko 2009 |
| Buryats | 419 | 34.8 | 0.7 | 32.0 | 2.1 | Derenko et al. 2000; 2003; 2007 |
| Kalmyks | 110 | 29.1 | 1.8 | 21.8 | 5.5 | Derenko et al. 2007 |
| Sojots | 30 | 46.7 | 0.0 | 46.7 | 0.0 | Derenko et al. 2003 |
| Khamnigans | 99 | 31.3 | 1.0 | 28.3 | 2.0 | Derenko et al. 2007 |
| **Western Siberia:** | 854 | 17.0 | 0.2 | 16.4 | 0.4 |  |
| Dolgans | 157 | 39.5 | 1.3 | 37.6 | 0.6 | Tamm et al. 2007 |
| Kets | 104 | 2.9 | 0.0 | 2.9 | 0.0 | Derbeneva et al. 2002a; Tamm et al. 2007 |
| Mansi | 161 | 12.4 | 0.0 | 11.8 | 0.6 | Derbeneva et al. 2002b; Pimenoff et al. 2008 |
| Khants | 106 | 16.0 | 0.0 | 16.0 | 0.0 | Pimenoff et al. 2008 |
| Nganasans | 78 | 28.2 | 0.0 | 26.9 | 1.3 | Derbeneva et al. 2002a; Goltsova et al. 2005 |
| Nenets | 58 | 22.4 | 0.0 | 22.4 | 0.0 | Saillard et al. 2000a |
| Nenets Tundra | 70 | 5.7 | 0.0 | 5.7 | 0.0 | Tamm et al. 2007 |
| Selkups | 120 | 3.3 | 0.0 | 3.3 | 0.0 | Tamm et al. 2007 |
| **Eastern Asia:** | 5166 | 26.9 | 0.0 | 22.5 | 4.4 |  |
| Ainu | 51 | 17.6 | 0.0 | 13.7 | 3.9 | Horai et al. 1996 |
| Koreans | 1297 | 33.3 | 0.0 | 28.8 | 4.5 | Horai et al. 1996; Tanaka et al. 2004; Lee et al. 2006; Derenko et al. 2007 |
| Mongolians | 150 | 22.7 | 0.0 | 20.7 | 2.0 | Kolman et al. 1998; Derenko et al. 2007 |
| Japanese | 1312 | 37.5 | 0.0 | 32.7 | 4.8 | Tanaka et al. 2004 |
| Daurs, China | 45 | 24.4 | 0.0 | 15.5 | 8.9 | Kong et al. 2003 |
| Kazakhs, China | 53 | 13.2 | 0.0 | 13.2 | 0.0 | Yao et al. 2004 |
| Chinese | 1930 | 16.4 | 0.0 | 12.4 | 4.0 | Horai et al. 1996; Kivisild et al. 2002; Yao et al. 2002; 2003; Metspalu et al. 2004; Wen et al. 2004 |
| Koreans, China | 48 | 33.3 | 0.0 | 22.9 | 10.4 | Kong et al. 2003 |
| Mongolians, China | 97 | 29.9 | 0.0 | 25.8 | 4.1 | Kong et al. 2003; Yao et al. 2004 |
| Orochens, China | 44 | 43.2 | 0.0 | 31.8 | 11.4 | Kong et al. 2003 |
| Uighurs, China | 47 | 10.6 | 0.0 | 6.4 | 4.2 | Yao et al. 2004 |
| Hui, China | 45 | 15.5 | 0.0 | 13.3 | 2.2 | Yao et al. 2004 |
| Evenks, China | 47 | 31.9 | 0.0 | 25.5 | 6.4 | Kong et al. 2003 |
| **Southeastern Asia:** | 1481 | 8.9 | 0.0 | 6.6 | 2.3 |  |
| Thai | 552 | 16.8 | 0.0 | 15.9 | 0.9 | Matspalu et al. 2004 |
| Island Southeastern Asia | 929 | 4.2 | 0.0 | 1.1 | 3.1 | Hill et al. 2007 |
| **Central Asia:** | 1386 | 16.6 | 0.1 | 15.3 | 1.2 |  |
| Kazakhs | 511 | 20.0 | 0.2 | 18.8 | 1.0 | Comas et al. 1998; Chaix et al. 2007; Tamm et al. 2007 |
| Karakalpaks | 108 | 17.6 | 0.0 | 14.8 | 2.8 | Chaix et al. 2007 |
| Kirghiz | 200 | 17.0 | 0.0 | 16.5 | 0.5 | Comas et al. 1998; Tamm et al. 2007 |
| Tajiks | 82 | 4.9 | 0.0 | 3.7 | 1.2 | Derenko et al. 2007; Tamm et al. 2007 |
| Turkmens | 178 | 16.3 | 0.0 | 16.3 | 0.0 | Malyarchuk 2002; Quintana-Murci et al. 2004; Chaix et al. 2007 |
| Uzbeks | 130 | 13.8 | 0.0 | 11.5 | 2.3 | Quintana-Murci et al. 2004; Chaix et al. 2007 |
| Uighurs | 177 | 13.6 | 0.0 | 11.3 | 2.3 | Comas et al. 1998; Tamm et al. 2007 |
| **Western Asia:** | 1440 | 1.4 | 0.0 | 1.1 | 0.3 |  |
| Turks | 268 | 1.9 | 0.0 | 1.9 | 0.0 | Richards et al. 2000; Quintana-Murci et al. 2004 |
| Kurdish | 77 | 7.8 | 0.0 | 3.9 | 3.9 | Quintana-Murci et al. 2004; Derenko et al. 2007 |
| Pakistani | 577 | 0.3 | 0.0 | 0.3 | 0 | Metspalu et al. 2004; Quintana-Murci et al. 2004 |
| Persians | 518 | 1.4 | 0.0 | 1.2 | 0.2 | Metspalu et al. 2004; Derenko et al. 2007 |
| **Southern Asia:** | 2544 | 1.8 | 0.0 | 1.2 | 0.6 |  |
| India | 2544 | 1.8 | 0.0 | 1.2 | 0.6 | Metspalu et al. 2004 |
| **Europe:** | 5302 | 0.7 | 0.0 | 0.3 | 0.4 |  |
| Hungarians | 344 | 0.6 | 0.0 | 0.6 | 0.0 | Lahermo et al. 2000; Egyed et al. 2007 |
| Poles | 947 | 0.3 | 0.0 | 0.2 | 0.1 | Richards et al. 2000; Malyarchuk et al. 2002; Grzybowski et al. 2007 |
| Romanians | 360 | 0.0 | 0.0 | 0.0 | 0.0 | Egyed et al. 2007 |
| Russians | 1132 | 0.5 | 0.0 | 0.3 | 0.2 | Malyarchuk 2002; Orekhov et al. 1999; Richards et al. 2000; Malyarchuk and Derenko 2001; Malyarchuk et al. 2002; 2004; Belyaeva et al. 2003; Grzybowski et al. 2007 |
| Karelians | 512 | 3.7 | 0.0 | 0.2 | 3.5 | Lappalainen et al. 2008 |
| Baltic Region population | 376 | 1.1 | 0.0 | 0.3 | 0.8 | Richards et al. 2000 |
| Scandinavians | 316 | 0.3 | 0.0 | 0.3 | 0.0 | Richards et al. 2000 |
| Germans | 554 | 0.2 | 0.0 | 0.2 | 0.0 | Baasner et al. 1998; Lutz et al. 1998; 1999; Pfeiffer et al. 1999; Baasner and Madea 2000 |
| Czechs | 354 | 0.3 | 0.0 | 0.3 | 0.0 | Richards et al. 2000; Vanecek et al. 2004; Malyarchuk et al. 2006 |
| Croats | 407 | 0.5 | 0.0 | 0.5 | 0.0 | GenBank data |
| **Caucasus:** | 544 | 5.0 | 0.0 | 5.0 | 0.0 |  |
| Georgians | 139 | 2.2 | 0.0 | 2.2 | 0.0 | Tambets et al. 2000 |
| Northern Caucasians | 199 | 4.0 | 0.0 | 4.0 | 0.0 | Richards et al. 2000 |
| Nogays | 206 | 7.8 | 0.0 | 7.8 | 0.0 | Bermisheva et al. 2005 |
| **Volga-Ural Region:** | 1323 | 4.8 | 0.0 | 4.6 | 0.2 |  |
| Bashkirs | 207 | 9.7 | 0.0 | 9.7 | 0.0 | Bermisheva et al. 2002 |
| Komis | 121 | 1.7 | 0.0 | 1.7 | 0.0 | Bermisheva et al. 2002 |
| Maris | 234 | 1.3 | 0.0 | 0.4 | 0.9 | Bermisheva et al. 2002; Orekhov 2002 |
| Mordvins | 120 | 0.8 | 0.0 | 0.8 | 0.0 | Bermisheva et al. 2002 |
| Tatars | 310 | 2.9 | 0.0 | 2.9 | 0.0 | Bermisheva et al. 2002; Orekhov 2002 |
| Udmurts | 189 | 12.7 | 0.0 | 12.7 | 0.0 | Bermisheva et al. 2002 |
| Chuvashis | 142 | 2.8 | 0.0 | 2.8 | 0.0 | Bermisheva et al. 2002 |

Note. References for population data are as in the Table S5. Note. Population subdivision of D sequences into subhaplogroups was performed based on specific nucleotide motifs: 16129A-16271C for D2; 3010A for D4 and 10397G, 16189C, 150T for D5.
