# Supplementary material for: Origin and Post-Glacial Dispersal of Mitochondrial DNA Haplogroups C and D in Northern Asia
Source: PLoS One. 2010 Dec 21;5(12):e15214. doi: 10.1371/journal.pone.0015214 (PMC3006427; doi:10.1371/journal.pone.0015214)
Supplement: Table S3 — Age estimates of haplogroup C subclusters calculated using different mutation rates. (DOC) [file pone.0015214.s005.doc]

Table S3. Age estimates of haplogroup C subclusters calculated using different mutation rates.

| Clade | No. of mtDNAs | Age estimates in ky | |
| --- | --- | --- | --- |
|  |  | complete genome rate (95% CI)^a^ | synonymous rate (± s.e.)^b^ |
| C | 174 | 27.37 (19.55; 35.44) | 26.33 ± 6.58 |
| >C1 | 4 | 16.88 (5.69; 28.71) | 9.86 ± 8.13 |
| >>C1a | 4 | 8.57 (2.6; 14.75) | 1.97 ± 1.97 |
| >C4 | 109 | 20.54 (14.32; 26.94) | 22.13 ± 6.38 |
| >>C4a’b’c | 106 | 20.8 (14.41; 27.39) | 22.39 ± 6.56 |
| >>>C4a | 63 | 25.27 (17.07; 33.77) | 19.77 ± 5.15 |
| >>>>C4a1 | 30 | 17.49 (9.93; 25.34) | 13.4 ± 3.8 |
| >>>>>C4a1a | 23 | 11.33 (6.09; 16.72) | 11.65 ± 4.17 |
| >>>>>>C4a1a1 | 7 | 5.6 (2.38; 8.87) | 5.63 ± 2.98 |
| >>>>>>C4a1a2 | 15 | 9.15 (4.59; 13.84) | 14.19 ± 6.2 |
| >>>>>>>C4a1a2a | 10 | 6.28 (3; 9.63) | 6.31 ± 3.7 |
| >>>>>>>>C4a1a2a1 | 4 | 4.55 (0.72; 8.49) | 5.91 ± 4.41 |
| >>>>>>>C4a1a2b | 3 | 7.89 (1.21; 14.85) | 5.26 ± 5.26 |
| >>>>>C4a1b | 5 | 11.71 (4.53; 19.18) | 7.88 ± 4.73 |
| >>>>C4a2 | 33 | 21.54 (12.6; 30.85) | 18.4 ± 5.77 |
| >>>>>C4a2a | 28 | 17.89 (9.43; 26.71) | 16.61 ± 6.51 |
| >>>>>>C4a2a1 | 12 | 6.1 (1.32; 11.03) | 5.26 ± 2.46 |
| >>>>>>C4a2a2 | 16 | 12.85 (4.25; 21.86) | 13.3 ± 7.52 |
| >>>>>>>C4a2a2a | 13 | 8.31 (1.19; 15.74) | 15.77 ± 9.24 |
| >>>>>>>>C4a2a2a1 | 12 | 5.88 (0.26; 11.7) | 8.54 ± 6.13 |
| >>>>>C4a2b | 5 | 14.49 (6.67; 22.63) | 20.5 ± 7.56 |
| >>>C4b | 43 | 7.64 (4.52; 10.82) | 6.78 ± 2.97 |
| >>>>C4b1 | 15 | 4.33 (1.89; 6.82) | 3.15 ± 1.82 |
| >>>>>C4b1a | 5 | 4.16 (1.26; 7.11) | 1.58 ± 1.58 |
| >>>>C4b2 | 4 | 1.29 (-0.49; 3.09) | 0 ± 0 |
| >>>>C4b3 | 6 | 5.66 (1.92; 9.49) | 7.88 ± 4.55 |
| >>>>C4b6 | 3 | 5.22 (1.03; 9.52) | 7.88 ± 4.55 |
| >C5 | 43 | 17.29 (11.41; 23.34) | 14.48 ± 4.68 |
| >>C5a | 18 | 10.31 (3.94; 16.91) | 10.51 ± 6.29 |
| >>>C5a1 | 10 | 2.85 (0.41; 5.32) | 0 ± 0 |
| >>>>C5a2 | 8 | 4.89 (-0.02; 9.95) | 3.94 ± 2.41 |
| >>>>>C5a2a | 7 | 2.59 (0.41; 4.79) | 4.51 ± 2.76 |
| >>C5b | 12 | 11.53 (5.86; 17.37) | 11.83 ± 5.65 |
| >>>>C5b1a | 7 | 3.71 (1.4; 6.05) | 3.38 ± 1.95 |
| >>>>C5b1b | 3 | 3.46 (-1.31; 8.38) | 5.26 ± 5.26 |
| >>C5c | 6 | 9.7 (3.17; 16.49) | 9.2 ± 4.74 |
| >>>C5c1 | 4 | 6.55 (1.08; 12.2) | 11.83 ± 6.83 |
| >>>>C5c1a | 3 | 5.22 (0.39;10.19) | 7.88 ± 4.55 |
| >>C5d | 6 | 11.53 (3.29; 20.15) | 14.45 ± 7.55 |
| >>>C5d1 | 5 | 2.06 (-0.41; 4.58) | 3.15 ± 3.15 |
| >C7 | 18 | 25.79 (15.35; 36.7) | 28.03 ± 8.98 |
| >>C7a | 14 | 22.51 (10.95; 34.69) | 28.16 ± 11.21 |
| >>>C7a1 | 14 | 16.78 (8.45; 25.46) | 20.27 ± 7.96 |
| >>>>C7a1a | 10 | 13.09 (5.04; 21.5) | 13.4 ± 6.92 |
| >>>>>C7a1a2 | 5 | 2.06 (0.04; 4.12) | 3.15 ± 2.23 |
| >>>>C7a1c | 3 | 7 (1.55; 12.63) | 7.88 ± 5.88 |

^a^Mutation rate is one mutation per every 3624 years (Soares et al. 2009);

^b^Mutation rate is one mutation per every 7884 years (Soares et al. 2009).
