# Supplementary material for: Origin and Post-Glacial Dispersal of Mitochondrial DNA Haplogroups C and D in Northern Asia
Source: PLoS One. 2010 Dec 21;5(12):e15214. doi: 10.1371/journal.pone.0015214 (PMC3006427; doi:10.1371/journal.pone.0015214)
Supplement: Table S4 — Age estimates of haplogroup D subclusters calculated using different mutation rates. (DOC) [file pone.0015214.s006.doc]

Table S4. Age estimates of haplogroup D subclusters calculated using different mutation rates.

| Clade | No. of mtDNAs | Age estimates in ky | |
| --- | --- | --- | --- |
|  |  | complete genome rate (95% CI)^a^ | synonymous rate (± s.e.)^b^ |
| D | 596 | 35.41 (25.59; 45.59) | 36.6 ± 7.88 |
| >D4 | 520 | 24.26 (20.23; 28.37) | 28.03 ± 4.07 |
| >>D4a | 52 | 10.5 (5.74; 15.4) | 9.55 ± 1.94 |
| >>>D4a1 | 41 | 6.39 (4.02; 8.79) | 6.54 ± 1.84 |
| >>>>D4a1a | 6 | 9.25 (2.56; 16.2) | 9.2 ± 4.74 |
| >>>>D4a1b | 12 | 3.24 (1.29; 5.21) | 3.94 ± 1.61 |
| >>>>D4a1c | 5 | 2.59 (-0.77; 6.01) | 1.58 ± 1.58 |
| >>>D4a2 | 5 | 8.43 (0.96; 16.25) | 11.04 ± 6.87 |
| >>>D4a3 | 4 | 17.59 (8.98; 26.57) | 27.59 ± 9.66 |
| >>D4b | 127 | 28.44 (19.26; 37.96) | 41.65 ± 9.68 |
| >>>D4b1 | 44 | 23.88 (16.22; 31.79) | 31.54 ± 7.28 |
| >>>>D3 | 6 | 2.15 (0.26; 4.06) | 2.63 ± 1.86 |
| >>>>D4b1a | 21 | 21.62 (12.72; 30.88) | 36.42 ± 10.85 |
| >>>>>D4b1a1 | 6 | 19.97 (7.63; 33.06) | 34.16 ± 14.75 |
| >>>>>D4b1a2 | 15 | 15.42 (7.71; 23.45) | 21.55 ± 8.46 |
| >>>>>>D4b1a2a | 14 | 12.18 (6.1; 18.47) | 13.52 ± 4.36 |
| >>>>>>>D4b1a2a1 | 10 | 10.61 (4.64; 16.79) | 11.04 ± 4.86 |
| >>>>>>>>D4b1a2a1a | 5 | 11.16 (4.08; 18.53) | 15.77 ± 7.4 |
| >>>>>>>>>D4b1a2a1a1 | 4 | 5.88 (2.01; 9.84) | 7.88 ± 3.94 |
| >>>>>>>>D4b1a2a1b | 5 | 4.69 (-1.15; 10.75) | 6.31 ± 6.31 |
| >>>>>>>D4b1a2a2 |  | 2.59 (-1.18; 6.44) | 0 ± 0 |
| >>>>D4b1b | 17 | 15.34 (5.98; 25.17) | 19.48 ± 9.48 |
| >>>>>D4b1b1 | 14 | 10.22 (2.22; 18.59) | 3.38 ± 1.59 |
| >>>>>D4b1b2 | 3 | 10.61 (2.79; 18.79) | 13.14 ± 7.88 |
| >>>D4b2 | 83 | 15.97 (9.62; 22.53) | 22.89 ± 6.72 |
| >>>>D4b2a | 22 | 15.02 (7.7; 22.63) | 21.14 ± 8.73 |
| >>>>>D4b2a1 | 6 | 1.72 (0.03; 3.42) | 1.31 ± 1.31 |
| >>>>>D4b2a2 | 15 | 10.98 (4.66; 17.52) | 13.14 ± 6.2 |
| >>>>>>D4b2a2a | 11 | 5.46 (1.92; 9.07) | 6.45 ± 2.38 |
| >>>>D4b2b’c | 58 | 12.65 (6.38; 19.14) | 23.24 ± 9.01 |
| >>>>>D4b2b | 54 | 9.8 (5.82; 13.88) | 15.18 ± 5.52 |
| >>>>>>D4b2b1 | 35 | 5.14 (3.36; 6.94) | 6.76 ± 1.69 |
| >>>>>>>D4b2b1a | 5 | 7.35 (2.07; 12.81) | 7.88 ± 3.53 |
| >>>>>>>D4b2b1c | 6 | 4.77 (1.93; 7.67) | 10.51 ± 3.72 |
| >>>>>>D4b2b2 | 5 | 9.52 (3.06; 16.22) | 9.46 ± 6.69 |
| >>>>>>D4b2b3 | 6 | 2.15 (0.26; 4.06) | 2.63 ± 1.86 |
| >>>>>>D4b2b5 | 3 | 11.53 (3.11; 20.35) | 10.51 ± 7.43 |
| >>>>>D4b2c | 4 | 3.24 (-0.96; 7.56) | 9.86 ± 6.54 |
| >>D4c | 32 | 21.62 (13.1; 30.48) | 15.03 ± 5.23 |
| >>>D4c1 | 21 | 19.56 (10.58; 28.93) | 8.26 ± 4.66 |
| >>>>D4c1a | 12 | 5 (2.85; 7.17) | 2.63 ± 1.31 |
| >>>>D4c1b | 9 | 12.14 (4.06; 20.59) | 5.26 ± 2.15 |
| >>>D4c2 | 11 | 9.12 (4.3; 14.08) | 12.18 ± 5.32 |
| >>D4e | 109 | 20.28 (11.8; 29.1) | 22.28 ± 7.76 |
| >>>D4e1’3 | 69 | 23.66 (11.68; 36.3) | 18.4 ± 8.65 |
| >>>>D4e1 | 68 | 21.04 (10.34; 32.29) | 18.55 ± 8.78 |
| >>>>>D2 | 56 | 15.37 (5.55; 25.7) | 11.12 ± 6.83 |
| >>>>>>D2a’b | 55 | 9.91 (3.54; 16.52) | 10.89 ± 6.95 |
| >>>>>>>D2a | 47 | 6.24 (1.34; 11.29) | 3.86 ± 1.8 |
| >>>>>>>>D2a1 | 33 | 5.06 (0.62; 9.62) | 2.87 ± 1.35 |
| >>>>>>>>>D2a1a | 26 | 1.88 (0.36; 3.42) | 2.73 ± 1.58 |
| >>>>>>>>>D2a1b | 6 | 2.15 (-0.08; 4.42) | 3.94 ± 2.94 |
| >>>>>>>>D2a2 | 9 | 1.14 (0.02; 2.27) | 1.75 ± 1.24 |
| >>>>>>>D2b | 8 | 10.27 (3; 17.85) | 5.91 ± 5.03 |
| >>>>>D4e1a | 11 | 11.11 (4.99; 17.44) | 2.15 ± 1.24 |
| >>>>D4e2 | 29 | 4.58 (2.96; 6.21) | 5.44 ± 1.63 |
| >>>>D4e4 | 7 | 8.28 (3.12; 13.6) | 5.63 ± 3.38 |
| >>>>>D4e4a | 4 | 7.22 (1.3; 13.36) | 0 ± 0 |
| >>>>D4e5 | 4 | 5.22 (1.58; 8.94) | 1.97 ± 1.97 |
| >>D4f | 10 | 13.93 (4.07; 24.33) | 11.04 ± 7.31 |
| >>>D4f1 | 9 | 3.46 (1.19; 5.76) | 4.38 ± 1.96 |
| >>D4g | 30 | 20.35 (11.66; 29.4) | 20.76 ± 6.65 |
| >>>D4g1 | 20 | 4.42 (1.96; 6.92) | 8.28 ± 3.23 |
| >>>D4g2 | 10 | 21.89 (13.34; 30.78) | 22.08 ± 6.78 |
| >>>>D4g2a | 7 | 16.58 (8.93; 24.52) | 16.89 ± 7.88 |
| >>D4h | 11 | 20.06 (12.51; 27.87) | 19.35 ± 6.04 |
| >>>D4h1 | 7 | 17.38 (8.5; 26.67) | 18.02 ± 7.96 |
| >>D4i | 10 | 10.61 (4.78; 16.64) | 0.79 ± 0.79 |
| >>>D4i2 | 6 | 7.44 (0.62; 14.56) | 1.31 ± 1.31 |
| >>D4j | 76 | 16.21 (10.6; 21.97) | 17.84 ± 5.11 |
| >>>D4j1 | 31 | 22.83 (10.49; 35.87) | 27.72 ± 12.18 |
| >>>>D4j1a | 27 | 6.5 (3.05; 10.03) | 6.42 ± 2.74 |
| >>>>>D4j1a1 | 6 | 4.77 (-0.11; 9.82) | 5.26 ± 3.22 |
| >>>>>D4j1a2 | 10 | 4.95 (-0.7; 10.81) | 4.73 ± 4.73 |
| >>>>D4j1b | 4 | 14.07 (5.72; 22.79) | 3.94 ± 3.94 |
| >>>D4j2 | 3 | 4.33 (-0.16; 8.96) | 7.88 ± 5.88 |
| >>>D4j3 | 5 | 14.49 (7.27; 21.98) | 20.5 ± 7.23 |
| >>>D4j4 | 3 | 3.46 (-0.68; 7.71) | 2.63 ± 2.63 |
| >>>D4j7 | 3 | 6.1 (0.43; 11.99) | 5.26 ± 5.26 |
| >>>D4j8 | 6 | 3.02 (0.21; 5.88) | 5.26 ± 3.22 |
| >>>D4j9 | 5 | 3.11 (0.23; 6.04) | 0 ± 0 |
| >>>D4j10 | 6 | 1.29 (-1.22; 3.84) | 3.94 ± 3.94 |
| >>D4k’o’p’ | 22 | 18.75 (11.71; 26.04) | 18.28 ± 6.32 |
| >>>D4k | 3 | 5.22 (0.39; 10.19) | 0 ± 0 |
| >>>D4o | 13 | 19.89 (11.63; 28.48) | 17.59 ± 6.62 |
| >>>>D4o1 | 6 | 9.7 (4.44; 15.12) | 3.94 ± 2.28 |
| >>>>D4o2 | 6 | 5.66 (2.56; 8.82) | 6.57 ± 2.94 |
| >>>D4p | 4 | 3.24 (-0.12; 6.67) | 5.91 ± 3.41 |
| >>D4l | 8 | 16.17 (8.51; 24.14) | 6.9 ± 3.55 |
| >>>D4l1 | 4 | 13.37 (5.6; 21.48) | 7.88 ± 6.23 |
| >>>D4l2 | 4 | 5.22 (0.43; 10.15) | 5.91 ± 3.41 |
| >>D4m | 8 | 20.45 (10.71; 30.64) | 19.71 ± 6.97 |
| >>>D4m2 | 6 | 11.99 (4.86; 19.4) | 19.71 ± 8.41 |
| >>D4n | 6 | 5.66 (0.89; 10.57) | 2.63 ± 1.86 |
| >>D4q | 15 | 8.79 (2.44; 15.39) | 7.36 ± 4.58 |
| >>>D4q1 | 10 | 6.55(0.07;13.29) | 11.04 ± 6.87 |
| >D5’6 | 76 | 47.77 (32.11; 64.15) | 41.29 ± 11.38 |
| >>D5 | 69 | 36.6 (24.86; 48.84) | 31.76 ± 9.62 |
| >>>D5a’b | 62 | 31.38 (20.23; 43.01) | 24.54 ± 7.09 |
| >>>>D5a | 48 | 22.56 (13.47; 32.02) | 13.3 ± 2.92 |
| >>>>>D5a1 | 8 | 8.23 (2.82; 13.82) | 14.78 ± 7.44 |
| >>>>>>D5a1a | 6 | 6.55 (1.91; 11.32) | 9.2 ± 5.73 |
| >>>>>D5a2 | 36 | 16.64 (8.39; 25.25) | 12.7 ± 3.39 |
| >>>>>>D5a2a’b | 31 | 11.23 (5.86; 16.76) | 12.46 ± 3.87 |
| >>>>>>>D5a2a | 21 | 12.05 (6.74; 17.52) | 13.89 ± 4.26 |
| >>>>>>>>D5a2a1 | 13 | 10.82 (4.89; 16.96) | 12.13 ± 5.94 |
| >>>>>>>>>D5a2a1a1 | 9 | 4.92 (1.64; 8.27) | 5.26 ± 2.77 |
| >>>>>>>>D5a2a2 | 4 | 7.22 (1.58; 13.05) | 13.8 ± 6.54 |
| >>>>>>>D5a2b | 10 | 1.29 (0.16; 2.42) | 1.58 ± 1.11 |
| >>>>>D5a3 | 4 | 16.17 (6.28; 26.58) | 15.77 ± 8.81 |
| >>>>>>D5a3a | 3 | 3.46 (0.07; 6.92) | 2.63 ± 2.63 |
| >>>>D5b | 14 | 16.58 (9.58; 23.82) | 20.27 ± 5.57 |
| >>>>>D5b1 | 13 | 12.52 (7.59; 17.57) | 19.41 ± 5.88 |
| >>>>>>D5b1a | 4 | 6.55 (1.08; 12.2) | 5.91 ± 3.41 |
| >>>>>>D5b1b | 7 | 8.66 (3.88; 13.59) | 9.01 ± 4.21 |
| >>>D5c | 7 | 34.64 (17.53; 52.86) | 10.14 ± 7.03 |
| >>>>D5c1 | 6 | 15.7 (4.12; 28.0) | 1.31 ± 1.31 |
| >>D6 | 7 | 22.72 (13.77; 32.04) | 41.67 ± 10.14 |
| >>>D6a | 4 | 19.01 (9.15; 29.35) | 37.45 ± 12.92 |
| >>>D6c | 3 | 9.7 (3.4; 16.23) | 23.65 ± 8.72 |

^a^Mutation rate is one mutation per every 3624 years (Soares et al. 2009);

^b^Mutation rate is one mutation per every 7884 years (Soares et al. 2009).
