# Supplementary material for: Origin and Post-Glacial Dispersal of Mitochondrial DNA Haplogroups C and D in Northern Asia
Source: PLoS One. 2010 Dec 21;5(12):e15214. doi: 10.1371/journal.pone.0015214 (PMC3006427; doi:10.1371/journal.pone.0015214)
Supplement: Table S5 — Distribution of D4b1a2a1 HVS1 mtDNA sequences in populations of northern Asia and America. (DOC) [file pone.0015214.s007.doc]

Table S5. Distribution of D4b1a2a1 HVS1 mtDNA sequences in populations of northern Asia and America.

| HVS1 sequence | Distribution^a^ | References |
| --- | --- | --- |
| **D4b1a2a1a** | | |
| 16093-16173-16223-16319-16362 | Kalmyks (1/110), Koryaks (3/218), Siberian Eskimos (18/227), Chukchi (25/483), Bashkirs (3/207), Karakalpaks (1/108), Greenland Eskimos (13/401), Canadian Eskimos (12/96) | Shields et al. 1993; Voevoda et al. 1994; Schurr et al. 1999; Bermisheva et al. 2002; Helgason et al. 2006; Tamm et al. 2007; Chaix et al. 2007; Volodko et al. 2008; Derenko 2009 |
| 16093-16150-16173-16223-16319-16362 | Siberian Eskimos (2/176) | Shields et al. 1993; Voevoda et al. 1994; Volodko et al. 2008 |
| 16093-16173-16223-16234-16319-16362 | Siberian Eskimos (1/51) | Tamm et al. 2007 |
| 16093-16173-16223-16234-16255-16319-16362 | Chukchi (6/182) | Volodko et al. 2008 |
| 16093-16172-16173-16223-16255-16319-16362 | Chuvantsi (2/32) | Volodko et al. 2008 |
| 16093-16172-16173-16215-16223-16319-16362 | Altaians (8/449), Shors (1/82), Khakassians (3/185), Uighurs (2/122), Tubalars (1/72) | Starikovskaya et al. 2005; Tamm et al. 2007; Derenko 2009 |
| 16172-16173-16215-16223-16319-16362 | Kirghiz (1/95) | Comas et al. 1998 |
| **D4b1a2a1b** | | |
| 16129-16173-16223-16319-16362 | Khamnigans (3/99), Buryats (14/621), Mongolians (1/47), Barghuts (5/149), Uighurs (1/122), Russians (1/74), Tatars (4/310), Udmurts (13/189), Bashkirs(2/207) | Bermisheva et al. 2002; Malyarchuk 2002; Orekhov 2002; Tamm et al. 2007; Derenko 2009 |
| 16129-16173-16223-16319-16352-16362 | Belorussians (1/292) | Kushnerevich 2008 |
| 16093-16129-16173-16223-16319-16362 | Poles (1/90) | Richards et al. 2000 |
| 16093-16129-16173-16223-16265C-16319-16362 | Maris (1/234), Poles (1/90) | Bermisheva et al. 2002; Orekhov 2002; Richards et al. 2000 |
| 16129-16173-16223-16266-16319-16362 | Udmurts (6/189) | Bermisheva et al. 2002 |

^a^ number of individuals with specific HVS1 sequence relative to total sample size is shown in parentheses.

**Supplementary Reference List**

Andrews RM, Kubacka I, Chinnery PF, Lightowlers R, Turnbull D, Howell N (1999) Reanalysis and revision of the Cambridge reference sequence for human mitochondrial DNA. Nat Genet 23: 147.

Baasner A, Madea B (2000) Sequence polymorphisms of the mitochondrial DNA control region in 100 German Caucasians. J Forensic Sci 45: 1343-1348.

Baasner A, Schafer C, Junge A, Madea B (1998) Polymorphic sites in human mitochondrial DNA control region sequences: population data and maternal inheritance. Forensic Sci Int 98: 169-178.

Belyaeva O, Bermisheva M, Khrunin A, Slominsky P, Bebyakova N, et al. (2003) Mitochondrial DNA variations in Russian and Belorussian populations. Hum Biol 75: 647-660.

Bermisheva MA, Kutuev IA, Korshunova TI, Dubova NA, Villems R, Khusnutdinova E.K (2004) Phylogeografic analysis of mitochondrial DNA Nogays: the high level of mixture of maternal lineages from Eastern and Western Eurasia. Mol Biol (Moscow) 38: 617-624.

Bermisheva MA, Kutuev IA, Spitsyn VA, Villems R, Batyrova AZ, et al. (2005) Analysis of mitochondrial DNA variation in the population of Oroks. Genetika 41: 78-84.

Bermisheva M, Tambets K, Villems R, Khusnutdinova E (2002) Diversity of mitochondrial DNA haplotypes in ethnic populations of the Volga-Ural region of Russia. Mol Biol (Moscow) 36: 990-1001.

Chaix R, Quintana-Murci L, Hegay T, Hammer MF, Mobasher Z, et al. (2007) From social to genetic structures in central Asia. Curr Biol 17: 43-48.

Comas D, Calafell F, Mateu E, et al. (1998) Trading genes along the Silk Road: mtDNA sequences and the origin of central Asian populations. Am J Hum Genet 63: 1824-1838.

Derbeneva OA, Starikovskaia EB, Volod'ko NV, Wallace DC, Sukernik RI (2002a) Mitochondrial DNA variation in Kets and Nganasans and the early peoples of Northern Eurasia. Genetika 38: 1554-1560.

Derbeneva OA, Starikovskaya EB, Wallace DC, Sukernik RI (2002b) Traces of early Eurasians in the Mansi of northwest Siberia revealed by mitochondrial DNA analysis. Am J Hum Genet 70: 1009-1014.

Derbeneva OA, Sukernik RI, Volodko NV, Hosseini SH, Lott MT, Wallace DC (2002c) Analysis of mitochondrial DNA diversity in the Aleuts of the Commander Islands and its implications for the genetic history of Beringia. Am J Hum Genet 71: 415-421.

Derenko MV (2009) Molecular phylogeography of aboriginal northern Asians based on mitochondrial DNA variability data. Dr Sci Biol thesis. Magadan: Institute of Biological Problems of the North.

Derenko MV, Grzybowski T, Malyarchuk BA, et al. (2003) Diversity of mitochondrial DNA lineages in South Siberia. Ann Hum Genet 67: 391-411.

Derenko MV, Malyarchuk BA, Dambueva IK, Shaikhaev GO, Dorzhu CM, Nimaev DD, Zakharov IA (2000) Mitochondrial DNA variation in two South Siberian aboriginal populations: implications for the genetic history of North Asia. Hum Biol 72: 945-973.

Derenko M, Malyarchuk B, Grzybowski T, et al (2007) Phylogeographic analysis of mitochondrial DNA in northern Asian populations. Am J Hum Genet 81: 1025-1041.

Derenko MV, Shields GF (1997) Diversity of mitochondrial DNA nucleotide sequences in three groups of aboriginal inhabitants of Northern Asia. Mol Biol (Moscow) 31: 784-789.

Egyed B, Brandstätter A, Irwin JA, Pádár Z, Parsons TJ, Parson W (2007) Mitochondrial control region sequence variations in the Hungarian population: analysis of population samples from Hungary and from Transylvania (Romania). Forensic Sci Int Genet 1: 158-162.

Fedorova SA, Bermisheva MA, Villems R, Maksimova NR, Khusnutdinova EK (2003) Analysis of mitochondrial DNA haplotypes in Yakut population. Mol Biol (Moscow) 37: 643-653.

GenBank, <http://www.ncbi.nlm.nih.gov/Genbank/>

Goltsova TV, Osipova LP, Zhadanov SI, Villems R (2005) The effect of marriage migration on the genetic structure of the Taimyr Nganasan population: genealogical analysis inferred from MtDNA markers. Genetika 41: 954-965.

Grzybowski T, Malyarchuk BA, Derenko MV, Perkova MA, Bednarek J, Woźniak M (2007) Complex interactions of the Eastern and Western Slavic populations with other European groups as revealed by mitochondrial DNA analysis. Forensic Sci Int Genet 1: 141-147.

Helgason A, Pálsson G, Pedersen HS, Angulalik E, Gunnarsdóttir ED, Yngvadóttir B, Stefánsson K (2006) mtDNA variation in Inuit populations of Greenland and Canada: migration history and population structure. Am J Phys Anthropol 130: 123-134.

Hill C, Soares P, Mormina M, et al. (2007) A mitochondrial stratigraphy for island southeast Asia. Am J Hum Genet 80: 29-43.

Horai S, Murayama K, Hayasaka K, Matsubayashi S, Hattori Y, Fucharoen G, Harihara S, Park KS, Omoto K, Pan IH (1996) mtDNA polymorphism in East Asian Populations, with special reference to the peopling of Japan. Am J Hum Genet 59: 579-590.

Kivisild T, Tolk HV, Parik J, Wang Y, Papiha SS, Bandelt HJ, Villems R (2002) The emerging limbs and twigs of the East Asian mtDNA tree. Mol Biol Evol 19: 1737-1751.

Kolman CJ, Sambuughin N, Bermingham E (1996) Mitochondrial DNA analysis of Mongolian populations and implications for the origin of New World founders. Genetics 142: 1321-1334.

Kong QP, Bandelt HJ, Sun C, et al. (2006) Updating the East Asian mtDNA phylogeny: A prerequisite for the identification of pathogenic mutations. Hum Mol Genet 15: 2076-2086.

Kong QP, Yao YG, Sun C, Bandelt HJ, Zhu CL., Zhang YP (2003) Phylogeny of East Asian mitochondrial DNA linerages inferred from complete sequences. Am J Hum Genet. 73: 671-676.

Kushnerevich EI (2008) Polymorphism of nucleotide sequences of mitochondrial DNA and Y-chromosome of modern native population of Belarus. PhD thesis. Minsk: Institute of Genetics and Cytology.

Lahermo P, Laitinen V, Sistonen P, Béres J, Karcagi V, Savontaus ML (2000) MtDNA polymorphism in the Hungarians: comparison to three other Finno-Ugric-speaking populations. Hereditas 132: 35-42.

Lappalainen T, Laitinen V, Salmela E, Andersen P, Huoponen K, Savontaus ML, Lahermo P (2008) Migration waves to the Baltic Sea region. Ann Hum Genet 72: 337-348.

Lee HY, Yoo JE, Park MJ, Chung U, Shin KJ (2006) Mitochondrial DNA control region sequences in Koreans: identification of useful variable sites and phylogenetic analysis for mtDNA data quality control. Int J Legal Med 120: 5-14.

Lutz S, Weisser HJ, Heizmann J, Pollak S (1998) Location and frequency of polymorphic positions in the mtDNA control region of individuals from Germany. Int J Legal Med 111: 67-77.

Lutz S, Weisser HJ, Heizmann J, Pollak S (1999) Erratum. Location and frequency of polymorphic positions in the mtDNA control region of individuals from Germany. Int J Legal Med 112: 145-150.

Malyarchuk BA (2002) Human mitochondrial genome variability with implication to genetic history of Slavs. Dr Sci Biol thesis. Magadan: Institute of Biological Problems of the North.

Malyarchuk BA, Derenko MV (2001) Mitochondrial DNA variability in Russians and Ukrainians: implication to the origin of the Eastern Slavs. Ann Hum Genet 65: 63-78.

Malyarchuk B, Derenko M, Grzybowski T, Lunkina A, Czarny J, Rychkov S, Morozova I, Denisova G, Miścicka-Sliwka D (2004) Differentiation of mitochondrial DNA and Y chromosomes in Russian populations. Hum Biol 76: 877-900.

Malyarchuk BA, Grzybowski T, Derenko MV, Czarny J, Miścicka-Sliwka D (2006) Mitochondrial DNA diversity in the Polish Roma. Ann Hum Genet 70: 195-206.

Malyarchuk BA, Grzybowski T, Derenko MV, Czarny J, Woźniak M, Miścicka-Sliwka D (2002) Mitochondrial DNA variability in Poles and Russians. Ann Hum Genet 66: 261-283.

Metspalu M, Kivisild T, Metspalu E, et al. (2004) Most of the extant mtDNA boundaries in south and southwest Asia were likely shaped during the initial settlement of Eurasia by anatomically modern humans. BMC Genet 5: 26.

Nasidze I, Ling EY, Quinque D, et al. (2004) Mitochondrial DNA and Y-chromosome variation in the Caucasus. Ann Hum Genet 68: 205-221.

Nasidze I, Quinque D, Dupanloup I, Rychkov S, Naumova O, Zhukova O, Stoneking M (2004) Genetic evidence concerning the origins of South and North Ossetians. Ann Hum Genet 68: 588-599.

Nasidze I, Quinque D, Ozturk M, Bendukidze N, Stoneking M (2005) MtDNA and Y-chromosome variation in Kurdish groups. Ann Hum Genet 69: 401-412.

Nasidze I, Stoneking M (2001) Mitochondrial DNA variation and language replacements in the Caucasus. Proc Biol Sci 268: 1197-1206.

Orekhov VA (2002) Characteristic of mitotypes of three ethnical groups of European part of Russia. PhD thesis. Moscow Russia: Vavilov Institute of General Genetics.

Orekhov V, Poltoraus A, Zhivotovsky LA, Spitsyn V, Ivanov P, Yankovsky N (1999) Mitochondrial DNA sequence diversity in Russians. FEBS Let 445: 197-201.

Pfeiffer H, Brinkmann B, Hühne J, Rolf B, Morris AA, Steighner R, Holland MM, Forster P (1999). Expanding the forensic German mitochondrial DNA control region database: genetic diversity as a function of sample size and microgeography. Int J Legal Med 112: 291-298.

Pimenoff VN, Comas D, Palo JU, Vershubsky G, Kozlov A, Sajantila A (2008) Northwest Siberian Khanty and Mansi in the junction of West and East Eurasian gene pools as revealed by uniparental markers. Eur J Hum Genet 16: 1254-1264.

Quintana-Murci L, Chaix R, Wells RS, et al. (2004) Where west meets east: the complex mtDNA landscape of the southwest and Central Asian corridor. Am J Hum Genet 74: 827-845.

Richards M, Macaulay V, Hickey E, et al. (2000) Tracing European founder lineages in the Near Eastern mtDNA pool. Am J Hum Genet 67: 1251-1276.

Saillard J, Evseeva I, Tranebjaerg L, Nørby S (2000a) Mitochondrial DNA diversity among Nenets. In: Renfrew C, Boyle K, editors. Archaeogenetics: DNA and the population prehistory of Europe. Cambridge: McDonald Institute for Archaeological Research. pp. 255-258.

Saillard J, Forster P, Lynnerup N, Bandelt HJ, Norby S (2000b) MtDNA variation among Greenland Eskimos: the edge of the Beringian expansion. Am J Hum Genet 67: 718-726.

Schurr TG, Sukernik RI, Starikovskaya YB, Wallace DC (1999) Mitochondrial DNA variation in Koryaks and Itel'men: population replacement in the Okhotsk Sea-Bering Sea region during the Neolithic. Am J Phys Anthropol 108: 1-39.

Shields GF, Schmiechen AM, Frazier BL, Redd A, Voevoda MI, Reed JK, Ward RH (1993) mtDNA sequences suggest a recent evolutionary divergence for Beringian and northern North American populations. Am J Hum Genet 53: 549-562.

Soares P, Ermini L, Thomson N, et al. (2009) Correcting for purifying selection: an improved human mitochondrial molecular clock. Am J Hum Genet 84:740-759.

Starikovskaya YB, Sukernik RI, Derbeneva OA, et al. (2005) Mitochondrial DNA diversity in indigenous populations of the southern extent of Siberia, and the origins of native American haplogroups. Ann Hum Genet 69: 67-89.

Starikovskaya YB, Sukernik RI, Schurr TG, Kogelnik AM, Wallace DC (1998) mtDNA diversity in Chukchi and Siberian Eskimos: implications for the genetic history of Ancient Beringia and the peopling of the New World. Am J Hum Genet 63: 1473-91.

Tambets K, Kivisild T, Metspalu E, et al. (2000) The topology of the maternal lineages of the Anatolian and Trans-Caucasus populations and the peopling of Europe: some preliminary considerations. In: Renfrew C, Boyle K, editors. Archaeogenetics: DNA and the population prehistory of Europe. Cambridge: McDonald Institute for Archaeological Research. pp. 219-235.

Tamm E, Kivisild T, Reidla M, et al. (2007) Beringian standstill and spread of Native American founders. PLoS One 2: e829.

Tanaka M, Cabrera VM, Gonzalez AM, et al. (2004) Mitochondrial genome variation in Eastern Asia and the peopling of Japan. Genome Res 14: 1832-1850.

Vanecek T, Vorel F, Sip M (2004) Mitochondrial DNA D-loop hypervariable regions: Czech population data. Int J Legal Med 118: 14-18.

Voevoda MI, Avksentyuk AV, Ivanova AV, Astakhova TI, Babenko VN, Kurilovich SA, Duffy LK, Segal B, Shields GF (1994) Molecular-genetic study of native population of Chukotka. Analysis of mitochondrial DNA and alcohol-metabolizing enzymes polymorphism. Sib Ecol J 2: 149-162.

Volodko NV, Starikovskaya EB, Mazunin IO, Eltsov NP, Naidenko PV, Wallace DC, Sukernik RI (2008) Mitochondrial genome diversity in arctic Siberians, with particular reference to the evolutionary history of Beringia and Pleistocenic peopling of the Americas. Am J Hum Genet 82: 1084-1100.

Wen B, Li H, Gao S, et al. (2005) Genetic structure of Hmong-Mien speaking populations in East Asia as revealed by mtDNA lineages. Mol Biol Evol 22: 725-734.

Yao YG, Kong QP, Bandelt HJ, Kivisild T, Zhang YP (2002) Phylogeographic differentiation of mitochondrial DNA in Han Chinese. Am J Hum Genet 70: 635-651.

Yao YG, Kong QP, Wang CY, Zhu CL, Zhang YP (2004) Different matrilineal contributions to genetic structure of ethnic groups in the Silk Road region in China. Mol Biol Evol 21: 2265-2280.
