# Supplementary material for: Origin and Post-Glacial Dispersal of Mitochondrial DNA Haplogroups C and D in Northern Asia
Source: PLoS One. 2010 Dec 21;5(12):e15214. doi: 10.1371/journal.pone.0015214 (PMC3006427; doi:10.1371/journal.pone.0015214)
Supplement: Table S6 — Control-region variation of the completely sequenced mtDNAs belonging to haplogroups C and D. (DOC) [file pone.0015214.s008.doc]

Table S6. Control-region variation of the completely sequenced mtDNAs belonging to haplogroups C and D.

| HG | HVS1 | HVS2 | ID | Population |
| --- | --- | --- | --- | --- |
| C4* | 223 287 298 327 | 73 189 249D 263 309.1C 309.2C 315.1C | Rus 184 | Russian |
| C4a1* | 093 223 298 327 | 73 249D 263 309.1C 315.1C | Br 338 | Buryat |
| C4a1* | 093 223 234 298 327 | 73 249D 263 315.1C | Tel 21 | Teleut |
| C4a1a1 | 129 150 223 298 327 | 73 195 249D 263 315.1C | Alt 148 | Altaian-Kizhi |
| C4a1a1 | 129 223 298 327 | 73 195 249D 263 315.1C | Br 318 | Buryat |
| C4a1a1 | 129 150 223 249 298 327 | 73 195 249D 263 315.1C | Br 518 | Buryat |
| C4a1a1 | 129 150 223 298 327 | 73 195 249D 263 315.1C | Bt 17 | Barghut |
| C4a1a1a | 129 150 223 298 327 | 73 195 249D 263 315.1C | Sh 28 | Shor |
| C4a1a1a | 086 129 150 223 298 327 | 73 195 249D 263 315.1C | Tel 24 | Teleut |
| C4a1a2 | 093 111 129 223 298 327 | 73 152 195 249D 263 309.1C 315.1C | Bt 58 | Barghut |
| C4a1a2 | 93 129 223 298 327 | 73 195 249D 263 309.1C 315.1C | Rus 193 | Russian |
| C4a1a2a | 093 129 223 298 327 | 73 195 249D 263 309.1C 315.1C | Alt 139 | Altaian-Kizhi |
| C4a1a2a | 093 129 223 298 311 327 | 73 195 249D 263 315.1C | Alt 150 | Altaian-Kizhi |
| C4a1a2a1 | 093 129 223 298 327 | 73 195 249D 263 309.1C 309.2C 315.1C | Br 339 | Buryat |
| C4a1a2a1 | 093 129 223 298 327 399 | 73 195 249D 263 309.1C 309.2C 315.1C | Bt 64 | Barghut |
| C4a1a2a2 | 129 223 293 298 327 | 73 195 249D 263 309.1C 315.1C | Bt 135 | Barghut |
| C4a1a2b | 093 223 298 327 | 73 249D 263 315.1C | Br 398 | Buryat |
| C4a1a2b | 093 129 213 223 298 327 | 73 195 249D 315.1C | Bt 88 | Barghut |
| C4a1a2b | 093 129 213 223 298 327 | 73 195 249D 315.1C | Hm 23 | Khamnigan |
| C4a2a1 | 171 223 298 327 344 357 | 47 73 249D 263 315.1C | Hm 29 | Khamnigan |
| C4a2a1 | 171 223 244 298 327 344 357 | 47 73 249D 263 315.1C | Br 610 | Buryat |
| C4a2a1 | 171 223 278 298 327 344 357 | 64 73 150 249D 263 315.1C | Alt 186 | Altaian-Kizhi |
| C4a2a1 | 171 223 298 327 344 357 | 73 249D 263 315.1C | Br 450 | Buryat |
| C4a2a1 | 171 223 298 327 344 357 | 73 195 249D 263 315.1C | Bt 60 | Barghut |
| C4a2a1 | 171 223 224 298 327 344 357 | 73 249D 263 315.1C | Evk 56 | Evenk |
| C4a2a1 | 171 223 298 327 344 357 | 73 249D 263 315.1C | Sh 34 | Shor |
| C4a2a1 | 171 223 298 327 344 357 | 73 249D 263 315.1C | Sh 35 | Shor |
| C4a2a2* | 189 223 298 327 357 | 47 73 249D 263 309.1С 315.1С | Bt 123 | Barghut |
| C4a2a2a1 | 223 239 327 357 | 47 73 207 249D 263 309.1C 315.1C | Tz 297G | Tajik |
| C4b* | 223 242 298 327 | 73 249D 263 309.1C 315.1C | Alt 165 | Altaian-Kizhi |
| C4b* | 223 298 327 | 73 249D 263 315.1C | Ev 31 | Even |
| C4b1* | 223 298 327 | 73 146 249D 263 309.1C 315.1C | Br 402 | Buryat |
| C4b1* | 223 298 327 | 73 146 234 249D 263 315.1C | Br 411 | Buryat |
| C4b1* | 223 270 298 327 | 73 146 249D 263 315.1C | Br 554 | Buryat |
| C4b1* | 189 223 298 327 | 73 146 249D 263 315.1C | Ev 21 | Even |
| C4b1* | 223 298 327 | 73 146 249D 263 309.1C 315.1C | Evk 41 | Evenk |
| C4b1* | 223 298 327 | 73 146 249D 263 309.1C 315.1C | Yak 38 | Yakut |
| C4b1* | 223 298 327 | 73 146 249D 263 309.1C 315.1C | Yak 39 | Yakut |
| C4b1a | 223 259.1A 294 298 327 | 73 146 249D 263 309.1C 315.1C | Br 379 | Buryat |
| C4b1a | 223 259.1A 298 327 | 73 146 249D 263 309.1C 315.1C | Bt 7 | Barghut |
| C4b2 | 124 223 298 318AT 327 | 73 249D 263 309.1C 315.1C | Krk 129 | Koryak |
| C4b3* | 223 291 298 327 | 73 249D 263 309.1C 315.1C | Alt 174 | Altaian-Kizhi |
| C4b3a | 223 291 298 327 | 73 152 249D 263 309.1C 315.1C | Br 534 | Buryat |
| C4b4 | 223 298 311 327 | 73 228 249D 263 309.1C 315.1C | Br 578 | Buryat |
| C4b4 | 223 298 311 327 | 73 189 249D 263 309.1C 315.1C | Hm 9 | Khamnigan |
| C4b5 | 223 298 327 | 73 249D 263 310 | Br 325 | Buryat |
| C4b5 | 223 298 327 | 73 249D 263 309.1C 309.2C 315.1C | Hm 45 | Khamnigan |
| C4b6 | 223 298 327 | 73 249D 263 309.1C 315.1C | Alt 202 | Altaian-Kizhi |
| C4b6 | 223 294 298 327 | 73 249D 263 309.1C 315.1C | Br 641 | Buryat |
| C4e | 223 298 327 | 73 151 152 249D 263 309.1C 315.1C | Sh 41 | Shor |
| C4e | 223 298 327 | 73 151 152 249D 263 309.1C 315.1C | Tl 10 | Teleut |
| C5a1 | 223 261 288 298 | 73 249D 263 315.1C | Alt 121 | Altaian-Kizhi |
| C5a1 | 093 223 261 288 298 | 57 73 249D 263 315.1C | Br 416 | Buryat |
| C5a1 | 223 261 288 298 | 57 73 249D 263 315.1C | Br 446 | Buryat |
| C5a1 | 223 261 288 298 | 73 249D 263 315.1C | Bt 14 | Barghut |
| C5a1 | 223 261 288 298 | 73 249D 263 309.1C 315.1C | Ev 29 | Even |
| C5a1 | 223 261 288 298 | 73 249D 263 309.1C 309.2C 315.1C | Ev 209G | Even |
| C5a1 | 223 261 288 298 | 73 249D 263 315.1C | Hm 82 | Khamnigan |
| C5a2* | 093 223 261 288 298 | 73 249D 263 315.1C | Br 308 | Buryat |
| C5a2a* | 093 189 223 261 288 298 | 73 249D 263 315.1C | Krk 117 | Koryak |
| C5a2a* | 093 189 223 261 288 298 | 73 249D 263 315.1C | Krk 123 | Koryak |
| C5a2a* | 093 189 223 261 288 298 | 73 189 249D 263 315.1C | Krk 130 | Koryak |
| C5a2a1 | 093 182AC189 193.1C 223 243 261 288 298 | 73 249D 263 315.1C | Krk 43 | Koryak |
| C5b1* | 189 193.1C 223 288 298 327 | 73 152 249D 263 310 | B 382 | Polish |
| C5b1* | 126 148 192 223 278 288 298 327 | 73 195 249D 263 315.1C | Br 369 | Buryat |
| C5b1a | 148 223 288 298 327 | 64 73 249D 263 309.1C 309.2C 315.1C | Alt 136 | Altaian-Kizhi |
| C5b1a | 148 223 288 294 298 327 | 73 249D 263 309.1C 309.2C 315.1C | Br 344 | Buryat |
| C5b1a | 148 223 288 298 327 | 73 249D 263 309.1C 309.2C 315.1C | Br 370 | Buryat |
| C5b1a | 148 223 288 298 311 327 | 73 249D 263 309.1C 309.2C 315.1C | Br 441 | Buryat |
| C5b1a | 148 223 257 288 298 327 343 | 73 249D 263 309.1C 315.1C | Evk 29 | Evenk |
| C5b1a | 094 148 223 288 298 327 | 73 249D 263 309.1C 315.1C | Hm 94 | Khamnigan |
| C5b1b | 148 164 223 288 298 327 | 73 249D 263 315.1C | Rus BgII-7 | Russian |
| C5b1b | 148 164 223 288 298 327 | 73 249D 263 315.1C | Br 540 | Buryat |
| C5b1b | 148 164 223 288 298 327 | 73 249D 263 315.1C | Yak 53 | Yakut |
| C5c1* | 093 223 234 288 298 327 | 54 73 249D 263 315.1C | B 96 | Polish |
| C5c1a | 093 223 234 288 298 327 | 73 249D 263 315.1C | Ser 162_06 | Polish |
| C5c1a | 093 223 234 288 298 327 | 73 152 195 249D 263 315.1C | Ser 37_08 | Polish |
| C5c2 | 093 223 288 291 298 | 73 249D 263 309.1C 315.1C | Tel 5888 | Teleut |
| C5d* | 093 148 223 284 288 298 327 | 73 207 249D 263 315.1C | Hm 19 | Khamnigan |
| C5d1 | 093 223 288 298 327 390 | 73 249D 263 315.1C | Alt 130 | Altaian-Kizhi |
| C7a1c | 223 298 327 | 73 146 249D 263 309.1C 315.1C | Evk 10 | Evenk |
| C7* | 051 093 145 223 298 311 327 | 73 152 249D 263 309.1C 315.1C | Bt 95 | Barghut |
| C7* | 183 223 248 298 327 | 73 143 152 225 249D 263 309.1C 315.1C | Kor 75 | Korean |
| D2b1* | 092 111 129 223 271 362 | 73 195 263 309.1C 315.1C | Km 80 | Kalmyk |
| D2b2 | 129 223 271 362 | 73 195 263 309.1C 315.1C | Bt 20 | Barghut |
| D2c | 129 223 362 | 73 263 310 | Br 639 | Buryat |
| D3 | 223 319 362 | 73 239 263 297 315.1C | Bt 42 | Barghut |
| D3 | 223 319 362 | 73 239 263 297 315.1C | Ev 16 | Even |
| D4* | 129 158 223 234 260 292 311 362 | 73 146 194 196 263 315.1C 385 | Kz 43 | Altaian Kazakh |
| D4a1* | 129 223 356 362 | 73 152 263 309.1C 315.1C | Bt 16 | Barghut |
| D4b1a1* | 223 319 362 | 73 309.1C 315.1C | Bt 44 | Barghut |
| D4b1a2a* | 189 223 319 362 | 73 263 315.1C | Hm 49 | Khamnigan |
| D4b1a2a1b | 129 173 223 319 362 | 73 183 263 309.1C 315.1C | Br 521 | Buryat |
| D4b1a2a1b | 129 173 223 319 362 | 73 183 263 309.1C 315.1C | Bt 115 | Barghut |
| D4b1a2a1b | 129 173 223 319 362 | 73 183 263 309.1C 315.1C | Bt 6 | Barghut |
| D4b1a2a1b | 129 173 223 319 362 | 73 183 263 309.1C 315.1C | Rus NN21 | Russian |
| D4b1a2a2 | 223 319 362 | 73 263 315.1C | Alt 170 | Altaian-Kizhi |
| D4b1a2a2 | 223 319 362 | 73 263 315.1C | Br 513 | Buryat |
| D4b2b* | 223 362 | 73 263 315.1C | Kor 20 | Korean |
| D4b2b1* | 223 362 | 73 194 263 309.1C 309.2C 315.1C | Br 569 | Buryat |
| D4b2b1c | 223 362 | 73 146 194 263 309.1C 315.1C | Kor 4 | Korean |
| D4b2b2* | 183AC 189 223 356 362 | 73 263 309.1C 309.2C 315.1C | Br 558 | Buryat |
| D4b2b5 | 223 335 362 | 73 263 279 315.1C | Br 361 | Buryat |
| D4b2b5 | 223 362 | 73 151 194 263 279 309.1C 315.1C | Bt 68 | Barghut |
| D4b2b5 | 223 335 362 | 73 263 279 315.1C | Bt 79 | Barghut |
| D4b2d | 223 287 362 | 73 263 309.1C 315.1C | Bt 10 | Barghut |
| D4b2d | 223 287 325 362 | 73 263 315.1C | Bt 97 | Barghut |
| D4c2a | 223 245 311 362 368 | 73 263 315.1C | Br 532 | Buryat |
| D4c2a | 223 245 311 362 368 | 73 263 315.1C | Bt 119 | Barghut |
| D4c2a | 223 245 311 362 368 | 73 263 315.1C | Hm 14 | Khamnigan |
| D4c2b | 223 245 362 | 73 263 315.1C | Bt 27 | Barghut |
| D4c2b | 093 164 223 245 362 | 73 152 263 315.1C | Bt 59 | Barghut |
| D4c2b | 223 245 362 | 73 208 263 315.1C | Rus 5154 | Russian |
| D4e1* | 188 223 362 | 73 263 309.1C 309.2C 315.1C | Cz IV-14 | Czech |
| D4e1a2a | 223 362 | 73 94 189 214 263 315.1 334 | Kor 23 | Korean |
| D4e4a | 223 291 362 | 73 263 309.1C 315.1C | Evk 45 | Evenk |
| D4e4a | 223 291 362 | 73 263 309.1C 315.1C | Evk 55 | Evenk |
| D4e4a | 189 223 362 | 73 263 309.1C 315.1C (453TA) | Evk 14 | Evenk |
| D4e4b | 138 223 362 | 73 196.1T 263 309.1C 315.1C | Rus Vo72 | Russian |
| D4e5 | 140 223 274 311 362 | 73 152 263 309.1 315.1 | Alt 193 | Altaian-Kizhi |
| D4e5 | 223 274 362 | 73 152 263 309.1C 309.2C 315.1C | Br 364 | Buryat |
| D4e5 | 173 223 249 274 291 362 | 73 152 263 315.1C | Bt 35 | Barghut |
| D4f1* | 223 362 | 73 152Y 263 315.1C | Bt 125 | Barghut |
| D4f1* | 067 223 362 | 73 263 315.1C | Kor 24 | Korean |
| D4g2a1b | 223 274 362 | 73 263 298 309.1C 315.1C | Bt 30 | Barghut |
| D4g2a1b | 223 274 362 | 73 263 298 309.1C 309.2C 315.1C | Bt 40 | Barghut |
| D4g2a1b | 223 274 362 | 73 263 298 309.1C 315.1C | Hm 24 | Khamnigan |
| D4g2b | 188 223 362 | 73 263 298 315.1C 316 | Br 355 | Buryat |
| D4h1* | 167 172 174 223 287 362 | 73 263 309.1C 315.1C | Bt 65 | Barghut |
| D4h4a | 093 223 311 362 | 73 151 152 153 263 309.1C 315.1C | Br 502 | Buryat |
| D4h4a | 223 311 362 | 73 152 263 315.1C | Bt 19 | Barghut |
| D4i2 | 223 294 362 | 73 146 195 263 315.1C | Bt 121 | Barghut |
| D4j* | 223 359 362 | 73 263 309.1C 315.1C | Br 360 | Buryat |
| D4j* | 223 362 | 73 263 315.1C | Bt 137 | Barghut |
| D4j* | 223 362 | 73 153 263 309.1C 309.2C 315.1C | Bt 53 | Barghut |
| D4j* | 223 286 362 | 73 263 309.1C 315.1C | Bt 105 | Barghut |
| D4j10 | 223 362 | 73 263 309.1C 315.1C | Br 515 | Buryat |
| D4j10 | 223 362 | 73 263 309.1C 315.1C | Bt 15 | Barghut |
| D4j10 | 223 362 | 73 263 309.1C 309.2C 315.1C | Hm 56 | Khamnigan |
| D4j1a* | 086 223 311 362 | 73 263 309.1C 315.1C | Br 524 | Buryat |
| D4j1a* | 086 223 362 | 73 152 263 315.1C | Br 528 | Buryat |
| D4j1a* | 086 150 223 274 362 | 73 263 315.1C | Bt 103 | Barghut |
| D4j1a* | 086 223 362 | 73 263 315.1C | Hm 84 | Khamnigan |
| D4j3b | 223 311 362 | 73 263 309.1C 315.1C | Br 573 | Buryat |
| D4j4 | 148 223 263 362 | 73 263 309.1C 315.1C | Br 613 | Buryat |
| D4j5 | 223 362 | 73 146 263 309.1C 315.1C | Br 313 | Buryat |
| D4j6 | 182 223 362 | 73 146 263 315.1C | Br 640 | Buryat |
| D4j7a | 082 223 362 | 73 263 315.1C | Br 526 | Buryat |
| D4j7a | 082 223 362 399 | 73 263 315.1C | Bt 74 | Barghut |
| D4j8 | 129 174 223 362 | 73 263 309.1C 309.2C 315.1C | Br 305 | Buryat |
| D4j8 | 174 223 362 | 73 263 309.1C 315.1C | Br 504 | Buryat |
| D4j8 | 129 174 223 362 | 73 263 309.1C 309.2C 315.1C | Bt 131 | Barghut |
| D4j8 | 111AC 174 223 362 | 73 263 309.1C 315.1C | Bt 98 | Barghut |
| D4j9 | 223 286 362 | 73 263 309.1C 315.1C | Bt 31 | Barghut |
| D4j9 | 223 286 362 | 73 263 309.1C 315.1C | Hm 85 | Khamnigan |
| D4j9 | 223 270 362 | 73 263 309.1C 315.1C | Br 599 | Buryat |
| D4j9 | 223 362 | 73 263 309.1C 315.1C | Bt 148 | Barghut |
| D4j9 | 223 362 | 73 263 309.1C 315.1C | Bt 72 | Barghut |
| D4k'o'p* | 223 362 | 73 152 195 263 309.1C 315.1C | Bt 4 | Barghut |
| D4k'o'p* | 223 362 | 73 152 195 263 309.1C 315.1C | Hm 17 | Khamnigan |
| D4l1* | 223 325 362 368 | 73 93 263 309.1C 315.1C | Bt 50 | Barghut |
| D4l2a | 223 274 362 368 | 125 127 263 309.1C 309.2C 315.1C | Evk 54 | Evenk |
| D4m2a* | 042 172 223 362 | 73 263 315.1C | Bt 2 | Barghut |
| D4m2a1 | 042 214 223 362 | 73 263 315.1C | Br 335 | Buryat |
| D4m2a1 | 042 093 214 223 362 | 73 263 315.1C | Evk 26 | Evenk |
| D4n* | 171AT 223 355CA 362 | 73 195 263 309.1C 309.2C 315.1C | Bt 146 | Barghut |
| D4o* | 223 290 362 | 73 183 263 315.1C | Br 571 | Buryat |
| D4o1* | 092 189 223 274 290 319 362 | 73 189 195 263 309.1C 315.1C | Hm 100 | Khamnigan |
| D4o1a | 183 223 274 290 319 362 | 73 195 263 309.1C 315.1C | Br 501 | Buryat |
| D4o2 | 093 223 290 362 365 | 73 195 263 309.1C 315.1C | Bt 133 | Barghut |
| D4o2 | 093 223 232 290 362 | 73 195 263 309.1C 315.1C | Bt 149 | Barghut |
| D4p* | 223 362 | 73 195 198 263 309 315.1C | Alt 184 | Altaian-Kizhi |
| D4p* | 223 362 | 73 195 198 263 315.1C | Br 511 | Buryat |
| D5a2* | 172 182AC 183AC 189 223 266 362 | 73 150 263 315.1C | Br 350 | Buryat |
| D5a2a* | 164 172 182AC 183AC 189 223 266 362 | 73 150 263 309.1C 315.1C | Br 349 | Buryat |
| D5a2a1* | 093 164 182AC 183AC 189 193.1C 223 266 362 | 73 150 263 309.1C 309.2C 309.3C 315.1C | Br 438 | Buryat |
| D5a2a1a1 | 092 102 164 182AC 183AC 189 193.1C 223 266 362 | 44.1C 73 150 263 309.1C 309.2C 315.1C | B 122 | Polish |
| D5a2b | 092 172 182AC 183AC 189 223 266 362 | 73 150 263 315.1C | Br 346 | Buryat |
| D5a2b | 092 172 182AC 183AC 189 192 223 266 362 | 73 150 263 309.1C 315.1C | Bt 9 | Barghut |
| D5a2b | 092 172 182AC 183AC 189 223 266 362 | 73 150 263 309.1C 315.1C | Evk 31 | Evenk |
| D5a3* | 182AC 183AC 189 223 360 362 | 73 150 151G 263 309.1C 315.1C | Kor 1 | Korean |
| D5a3a | 126 136 182C 183C 189 193.1C 223 360 362 | 73 150 263 309.1C 315.1C | Rus VN25 | Russian |

Note. Variant positions from the rCRS (Andrews et al. 1999) are shown (minus 16000 for HVS1). These are transitions, transversions are further specified. The presence of insertions or deletions is indicated by .1, .2 and .3 or D, respectively, following the nucleotide position.
